# A kinetic flux vector splitting scheme for shallow water equations incorporating variable bottom topography and horizontal temperature gradients

**DOI:** 10.1371/journal.pone.0197500

**Published:** 2018-05-31

**Authors:** M. Rehan Saleem, Waqas Ashraf, Saqib Zia, Ishtiaq Ali, Shamsul Qamar

**Affiliations:** 1 COMSATS Institute of Information Technology, Park Road, Chak Shahzad Islamabad, Pakistan; 2 Institute of Space Technology, Islamabad, Pakistan; Duke University Marine Laboratory, UNITED STATES

## Abstract

This paper is concerned with the derivation of a well-balanced kinetic scheme to approximate a shallow flow model incorporating non-flat bottom topography and horizontal temperature gradients. The considered model equations, also called as Ripa system, are the non-homogeneous shallow water equations considering temperature gradients and non-uniform bottom topography. Due to the presence of temperature gradient terms, the steady state at rest is of primary interest from the physical point of view. However, capturing of this steady state is a challenging task for the applied numerical methods. The proposed well-balanced kinetic flux vector splitting (KFVS) scheme is non-oscillatory and second order accurate. The second order accuracy of the scheme is obtained by considering a MUSCL-type initial reconstruction and Runge-Kutta time stepping method. The scheme is applied to solve the model equations in one and two space dimensions. Several numerical case studies are carried out to validate the proposed numerical algorithm. The numerical results obtained are compared with those of staggered central NT scheme. The results obtained are also in good agreement with the recently published results in the literature, verifying the potential, efficiency, accuracy and robustness of the suggested numerical scheme.

## Introduction

In the recent years, the study of hyperbolic systems with source terms has attracted much attention in the field of computational fluid dynamics (CFD) due to their wide rang physical and engineering applications. One example of such systems is the set of Saint-Venant equations which are widely used in the ocean currents and hydraulic engineering to describe the hydraulic jumps and shallow water flows [1–3]. In the early decades many numerical methods have been developed to investigate such flows.

The present work deals with the numerical approximation of shallow water equations which have temperature fluctuations of prime interest [4]. This set of shallow water equations, containing horizontal temperature gradient terms, is called as Ripa system and is used to investigate ocean currents [5, 6]. The main difference between the well known shallow water model and the Ripa system is the definition of steady states at rest. Indeed, the Ripa system involves the steady states at rest governed by the system of partial differential equations. This system is a non-homogenous hyperbolic system with non-zero source terms which appear after incorporating horizontal temperature gradients in the shallow water equations [7–12].

The classical nonlinear shallow water equations were derived in 1871 by de Saint-Venant [13]. Currently these equations are widely used in practice and one can find thousands of publications devoted to the applications, validations and numerical solutions of these equations [14]. In the pioneering work of Savage and Hutter [15] a shallow water type model in one-space dimension has been proposed to study aerial avalanches, followed by an extension of their approach to two-space dimensions [16].

Several well-balanced and higher order accurate computational methods have been designed for solving Saint-Venant equations and Ripa system in one and two space dimensions. Some of the well known finite volume schemes for solving systems of conservation laws include the finite volume weighted essentially non-oscillatory (WENO) scheme, conservation element solution element (CESE) scheme, central upwind (CUP) scheme, central Nessyahu Tadmor (NT) scheme, as well as Bhatnagar Gross Krook (BGK) and kinetic flux vector splitting (KFVS) schemes [17–23]. In this work, the KFVS scheme is extended to solve the current shallow flow model equations.

In the literature, many positivity preserving and well-balanced schemes have been suggested for solving single and two phase shallow flow models [24–31]. An efficient computational scheme should preserve the positivity of *h* and *θ* and should capture the steady states and small perturbations of the solutions. In addition to the above requirements, a good numerical method should avoid developing spurious oscillations of pressure in the neighborhood of temperature jumps. Such type of typical oscillations may appear when a Godunov-type finite volume scheme is applied [32].

Zhou et al. have developed a well-balanced and robust numerical scheme based on the Harten Lax and van Leer (HLL) Riemann solver and surface gradient method (SGM) for interpolating the initial data [33]. The main advantage of their approach is the consideration of *h* + *B* for the initial reconstruction instead of *h* that guarantees the achievement of steady state. In this manuscript, the same *h* + *B* is used as a conservative variable. Audusse et al. have developed a first-order accurate KFVS scheme based on the collisionless Boltzmann transport equation for the system of shallow water equations [34]. That scheme is well-balanced based on the appropriate choice of flux splitting but the accuracy of that scheme was not sufficient for some test problems. Furthermore, for the shallow water equations the source terms effects the evolution around the cell interface and has tendency to change the flux terms. Thus, special attention is needed to the discretization of source terms. Our proposed KFVS scheme, which is second order accurate and discretizes the source terms appropriately, has the capability to overcome all such difficulties.

The Godunov upwind finite volume schemes are based on the hyperbolic structure of the underlying conservation laws [4, 35]. However, for some systems, it is difficult to find the explicit expressions of eigenvalues. Thus, the development of Riemann solvers are not possible for such systems. In such scenarios, the kinetic theory based numerical schemes can be very interesting if the Maxwellian distributions are available for such systems. One of the fascinating aspects of the kinetic schemes is that when applied to Euler equations of gas dynamics, they preserve the positivity of mass, density and pressure. As a result, the kinetic schemes are unconditionally stable in the *L*^1^-norm. Moreover, they also possess the entropy property as a consequence of the celebrated Boltzmann H-theorem [35]. Since the form of the numerical dissipation in the FVS scheme is consistent with the Navier-Stokes viscous terms, the robustness of the KFVS schemes can be easily understood, i.e., the absence of numerical shock instability. These advantages of the kinetic schemes are also very attractive when solving the current shallow flow model [31].

There are two types of gas kinetic schemes. One of the well-balanced gas kinetic schemes is KFVS scheme which is based on the collionless Boltzmann equation, while the other scheme is based on the BGK model [36, 37]. On combining the dynamical effects of both gas evolution and projection stages, the governing equations are physically same for both KFVS and BGK schemes. The major difference between both the two schemes is that the particle collision time *τ* in BGK scheme is replaced by the Courant Friedrichs Lewy (CFL) time step Δ*t* of the KFVS scheme [38]. As the structures of both Ripa system and shallow water model are very similar, we can expect the same performance of this well balanced scheme for the Ripa model. Furthermore, as the shallow water equations can be recovered by providing a constant temperature *θ*, the extended scheme for the Ripa system will also preserve the lake at rest steady states exactly for the constant *θ*.

The KFVS scheme is based on the Boltzmann equation of kinetic theory of fluids. Due to their intrinsic upwinding, multidimensional and free of Riemann solver properties, gas kinetic schemes have got increasing attention of the researchers in this field [39–42]. The current KFVS scheme has capability to tackle all difficulties associated with the non-conservative part of the equations. The basic idea behind this scheme is that it uses information of local propagation speeds and estimates solutions in the form of cell averages. Moreover, this scheme has an upwind nature, as it takes care about the direction of propagation of wave by measuring the one-sided local speeds. Apart from its advantages, the KFVS has some limitations as well. For example, the scheme strictly depends on the Boltzmann equation and Maxwellian distribution function. In the case of complications in the Maxwellian distribution function, the KFVS method becomes complicated and requires more computational effort. The application of KFVS is almost not possible in the absence of Maxwellian distribution function. This scheme has been successfully applied to approximate several models in gas dynamics. Mandal and Deshpande [43] have used this scheme to investigate bump in a channel problem and it was found that the explicit flux function of KFVS scheme was similar to the flux function of Van Leer [44]. For the simulation of two dimensional problems for structured and unstructured meshes, the high order KFVS schemes have been used [22, 45]. In the current KFVS scheme, we start with the cell average initial data of conserved variables and calculate the cell averaged values in the same cells at updated time step. In order to achieve second order accuracy of the scheme, MUSCL-type initial reconstruction procedure and Runge-Kutta time stepping method are used.

As particles have the ability to move in forward and backward directions with the fluid motion, the fluxes of conservative variables can be divided into forward and backward fluxes at the cell interface, i.e.,

$$\begin{array}{c} F_{i+1/2}=F^{+}\left(W_{i}\right)+F^{-}\left(W_{i+1}\right), \end{array}$$ (1)

where, *W*_*i*_ represents the vector of conserved variables within the cell [*x*_*i*−1/2_, *x*_*i*+1/2_] and *F*(*W*_*i*_) denotes the vector of flux functions. For validation, the results of our proposed scheme are compared with those obtained from the staggered central NT scheme [10, 12]. The main reason for choosing this scheme is its simplicity, efficiency, robustness, Riemann-solver free nature and second-order accuracy. The current KFVS scheme is developed in such a way that it preserves the positivity of physical variables like height, temperature and pressure. Thus, the scheme gives stable results.

The present article is organized as follows. Sections 2 and 3 are devoted to the introduction of one-dimensional Ripa system and to the derivation of KFVS scheme, respectively. The two-dimensional (2D) Ripa system and the corresponding 2D KFVS scheme are presented in the Sections 4 and 5, respectively. Numerous numerical case studies are carried out in Section 6. Finally, concluding remarks are given in Section 7.

## Single-phase one-dimensional Ripa model

Firstly, we consider the one-dimensional single-phase shallow flow model with variable bottom topography [5–7]

$$\begin{array}{c} \partial_{t}h+\partial_{x}\left(hu\right)=0\;, \end{array}$$ (2)

$$\begin{array}{cc} \partial_{t}\left(hu\right) & +\partial_{x}\left(hu^{2}+\frac{g}{2}h^{2}\theta \right)=-gh\theta \partial_{x}B\;, \end{array}$$ (3)

$$\begin{array}{cc} \partial_{t}\left(h\theta \right) & +\partial_{x}\left(uh\theta \right)=0\;, \end{array}$$ (4)

where *h* is the flow height, *g* is the gravitational acceleration constant, *u* is the flow velocity along the *x* direction, B=B(x),x∈R denotes the bottom topography from a given level and *θ* represents the temperature fluctuation function. The stationary steady states for this model are expressed as

$$\begin{array}{c} u=0,\;w:=h+B=\text{constant},\;\theta =\text{constant}. \end{array}$$ (5)

Eqs (2)–(4) can be written in the compact form as

$$\begin{array}{c} \frac{\partial W}{\partial t}+\frac{\partial F(W)}{\partial x}=\tau ,\; \end{array}$$ (6)

where *W* is the vector of conserved variables, *F* represents the vector of fluxes and *τ* denotes the source term. The conserved variables are give as

$$\begin{array}{c} w_{1}\overset{\text{def}}{=}h,\;w_{2}\overset{\text{def}}{=}hu,\;w_{3}\overset{\text{def}}{=}h\theta \;. \end{array}$$ (7)

and the conservative fluxes are given as

$$\begin{array}{c} f_{1}\overset{\text{def}}{=}hu,\;f_{2}\overset{\text{def}}{=}hu^{2}+\frac{g}{2}h^{2},\;f_{3}\overset{\text{def}}{=}uh\theta . \end{array}$$ (8)

Moreover, the non-conservative source terms are expressed as

$$\begin{array}{c} \tau_{1}\overset{\text{def}}{=}0,\;\tau_{2}\overset{\text{def}}{=}-gh\theta \partial_{x}B,\;\tau_{3}\overset{\text{def}}{=}0. \end{array}$$ (9)

Above equations can be expressed in quasi-linear form as

$$\begin{array}{c} \partial_{t}W+A\left(W\right)\partial_{x}\left(W\right)=R\left(W\right)\;, \end{array}$$ (10)

where *W* = (*w*_1_, *w*_2_, *w*_3_)^*T*^, *R*(*W*) = [0, −*ghθ*∂_*x*_ *B*, 0]^*T*^ and *A*(*W*) is the Jacobian matrix whose element at the *k*-th row and *l*-th column is $\frac{\partial f_{k}}{\partial w_{l}}$ for *k*, *l* = 1, 2, 3 and the functions *f*_*k*_(*w*_1_, *w*_2_) are defined in Eq (8). The matrix *A*(*W*) is given as

$$\begin{array}{c} A\left(W\right)=\left(\begin{array}{ccc} 0 & 1 & 0\\ -u^{2}+\frac{g}{2}h\theta & 2u & \frac{g}{2}h\\ -u\theta & \theta & u \end{array}\right). \end{array}$$ (11)

The eigenvalues of the Jacobian matrix A(W) are λ_1_ = *u*, $\lambda_{2}=u+\sqrt{gh\theta }$ and $\lambda_{3}=u-\sqrt{gh\theta }$. Since these eigenvalues are all real, so the one-dimensional Ripa system under consideration is hyperbolic.

## KFVS scheme for one-dimensional single-phase shallow flow model

The flux of conserved variables is related to the particle motion across cell interfaces. For the one-dimensional flow, the particle motion in this direction determines the flux function *F*(*W*).

In statistical mechanics, the distribution of moving particles in the *x*- direction can be considered by local Maxwellian distribution function. The Maxwellian distribution function in normal direction *n* ∈ {*x*} is given as [22, 46],

$$\begin{array}{c} f_{M}\left(t,n,v_{n}\right)=h{\left(\frac{\lambda }{\pi }\right)}^{\frac{1}{2}}\exp\left[-\lambda {\left(v_{n}-u_{n}\right)}^{2}\right],\;\;\lambda =\frac{1}{gh}\;, \end{array}$$ (12)

where *u*_*n*_ is the average fluid velocity in the *n*-direction, *v*_*n*_ is the individual particle velocity in the same direction, and λ is the normalization factor of the distribution of random velocity. The transport of any flow quantity is due to the movement of particles. Considering the distribution function *f*_*M*_ in Eq (12), particles can be split into two groups. One group move to the right side with positive velocity (*u*_*n*_ > 0) and the other group move to the left side with negative velocity (*u*_*n*_ < 0). Before splitting the fluxes, let us define

$$\begin{array}{c} {\left\langle v^{0}\right\rangle }_{n}=1=\int_{-\infty }^{\infty }{\left(\frac{\lambda }{\pi }\right)}^{\frac{1}{2}}e^{-\lambda {\left(v_{n}-u_{n}\right)}^{2}}dv_{n}\;, \end{array}$$ (13)

$$\begin{array}{c} {\left\langle v^{1}\right\rangle }_{n}=u=\int_{-\infty }^{\infty }{\left(\frac{\lambda }{\pi }\right)}^{\frac{1}{2}}v_{n}e^{-\lambda {\left(v_{n}-u_{n}\right)}^{2}}dv_{n}\;. \end{array}$$ (14)

The above two moments are sufficient to split all the fluxes. In order to simplify the notation, one can define [22]

$$\begin{array}{c} {\left\langle v^{0}\right\rangle }_{+n}=\int_{0}^{\infty }{\left(\frac{\lambda }{\pi }\right)}^{\frac{1}{2}}e^{-\lambda {\left(v_{n}-u_{n}\right)}^{2}}dv_{n}=\frac{1}{2}\text{erfc}\left(-\sqrt{\lambda }u_{n}\right)\;, \end{array}$$ (15)

$$\begin{array}{c} {\left\langle v^{0}\right\rangle }_{-n}=\int_{-\infty }^{0}{\left(\frac{\lambda }{\pi }\right)}^{\frac{1}{2}}e^{-\lambda {\left(v_{n}-u_{n}\right)}^{2}}dv_{n}=\frac{1}{2}\text{erfc}\left(\sqrt{\lambda }u_{n}\right)\;, \end{array}$$ (16)

and

$$\begin{array}{c} {\left\langle v^{1}\right\rangle }_{+n}=\int_{0}^{\infty }{\left(\frac{\lambda }{\pi }\right)}^{\frac{1}{2}}v_{n}e^{-\lambda {\left(v_{n}-u_{n}\right)}^{2}}dv_{n}=u_{n}{\left\langle v^{0}\right\rangle }_{+n}+\frac{1}{2}\frac{e^{-\lambda u_{n}^{2}}}{\sqrt{\pi \lambda }}\;, \end{array}$$ (17)

$$\begin{array}{c} {\left\langle v^{1}\right\rangle }_{-n}=\int_{-\infty }^{0}{\left(\frac{\lambda }{\pi }\right)}^{\frac{1}{2}}v_{n}e^{-\lambda {\left(v_{n}-u_{n}\right)}^{2}}dv_{n}=u_{n}{\left\langle v^{0}\right\rangle }_{-n}-\frac{1}{2}\frac{e^{-\lambda u_{n}^{2}}}{\sqrt{\pi \lambda }}\;. \end{array}$$ (18)

Here, the complementary error function is defined as

$$\begin{array}{c} \text{erfc}\left(x\right)=\frac{2}{\sqrt{\pi }}\int_{x}^{\infty }e^{-t^{2}}dt\;. \end{array}$$ (19)

In order to apply KFVS scheme, first we divide the domain of interest into *N* sub-domains. Let us define a cell *C*_*i*_ by interval $\left[x_{i-\frac{1}{2}},x_{i+\frac{1}{2}}\right]$ for *i* = 1, 2, ⋯, *N*. Therefore, $\Delta x=x_{i+\frac{1}{2}}-x_{i-\frac{1}{2}}$ represents the uniform width of cell, the points *x*_*i*_ = *iΔx* denote to the cells center and the points $x_{i\pm \frac{1}{2}}=x_{i}\pm \Delta x/2$ represent the cells faces. We start the process with a cell averaged initial data $W_{i}^{n}$ at time step *t*^*n*^ and compute the cell average updated solution $W_{i}^{n+1}$ over the same cell at the next time step *t*^*n*+1^.

Let us consider the one dimensional single-phase shallow flow model. Using the above relations, the flux functions in Eq (8) can be splitted as

$$\begin{array}{c} F\left(W_{i}\right)=F^{+}\left(W_{i}\right)+F^{-}\left(W_{i+1}\right)\;. \end{array}$$ (20)

Following [22], the flux vectors at the left and right interfaces of the cell *C*_*i*_ are given as

$$\begin{array}{c} F_{i-\frac{1}{2}}=F_{i-1}^{+}+F_{i}^{-},\;\;\;\;\;\;F_{i+\frac{1}{2}}=F_{i}^{+}+F_{i+1}^{-}\;, \end{array}$$ (21)

where, $F_{i}^{\pm }$ ≔ *F*^±^(*W*_*i*_). Thus we have,

$$\begin{array}{c} F^{\pm }={\left\langle v^{1}\right\rangle }_{\pm n}\left(\begin{array}{c} h\\ hu\\ h\theta \end{array}\right)+{\left\langle v^{0}\right\rangle }_{\pm n}\left(\begin{array}{c} 0\\ \frac{1}{2}gh^{2}\theta \\ 0 \end{array}\right)\;. \end{array}$$ (22)

Integration of Eq (6) over the cell $C_{i}:=\left[x_{i-\frac{1}{2}},x_{i+\frac{1}{2}}\right]$ gives the following semi-discrete kinetic upwind scheme

$$\begin{array}{c} \frac{dW_{i}}{dt}=-\frac{F_{i+\frac{1}{2}}-F_{i-\frac{1}{2}}}{\Delta x}-\frac{T_{i+\frac{1}{2}}-T_{i-\frac{1}{2}}}{\Delta x}\;, \end{array}$$ (23)

where, *W*_*i*_ denotes the cell averaged values of the conserved variables. Moreover, following [32] a modified Godunov type approach is taken into account for the discretization of the non-conservative term. In this approach, non-conservative terms appear on the right hand side are approximated as cell averaged values and the differential terms are approximated by using flux splitting technique,

$$\begin{array}{c} T_{i+\frac{1}{2}}-T_{i-\frac{1}{2}}=\left(\begin{array}{c} 0\\ -gh\theta [{\left(B\right)}_{i+\frac{1}{2}}-{\left(B\right)}_{i-\frac{1}{2}}]\\ 0 \end{array}\right)\;. \end{array}$$ (24)

Here, the cell averaged values *W*_*i*_ are defined as

$$\begin{array}{c} W_{i}:=W_{i}\left(t\right)=\frac{1}{\Delta x}\int_{x_{i-\frac{1}{2}}}^{x_{i+\frac{1}{2}}}W\left(t,x\right)dx\; \end{array}$$ (25)

and $F_{i+\frac{1}{2}}$ and $F_{i-\frac{1}{2}}$ are given by (21).

The above scheme is only first order accurate in space. To achieve high order accuracy, the initial reconstruction strategy must be applied for the interpolation of cell averaged variables *W*_*i*_. Here, a second order accurate MUSCL-type initial reconstruction procedure is employed. Starting with a piecewise constant solution *W*_*i*_, one can reconstructs a piecewise linear (MUSCL-type) approximation by selecting slope vector (differences) *W*^*x*^. Then, boundary extrapolated values are given as

$$\begin{array}{c} W_{i}^{LX}=W_{i}-\frac{1}{2}W_{i}^{x}\;,\;\;W_{i}^{RX}=W_{i}+\frac{1}{2}W_{i}^{x}\;. \end{array}$$ (26)

A possible computation of these slopes is given by the family of discrete derivatives parameterized with 1 ≤ *ϑ* ≤ 2, for example

$$\begin{array}{c} W_{i}^{x}=MM\left\{\vartheta \Delta W_{i+\frac{1}{2}},\frac{\vartheta }{2}\left(\Delta W_{i+\frac{1}{2}}+\Delta W_{i-\frac{1}{2}}\right),\vartheta \Delta W_{i-\frac{1}{2}}\right\}\;, \end{array}$$ (27)

where, Δ denotes central differencing and 1 ≤ *ϑ* ≤ 2 is a parameter. For *ϑ* = 1 the limiter is most dissipative and is least dissipative for *ϑ* = 2. Moreover,

$$\begin{array}{c} \Delta W_{i+\frac{1}{2}}=W_{i+1}-W_{i}\;, \end{array}$$ (28)

and *MM* denotes the min-mod nonlinear limiter

$$\begin{array}{c} MM\left\{x_{1},x_{2},...\right\}=\left\{ \begin{array}{c} {\text{min}}_{i}\left\{x_{i}\right\}\;\text{if}\;x_{i}>0\;\forall i\;,\\ {\text{max}}_{i}\left\{x_{i}\right\}\;\text{if}\;x_{i}<0\;\forall i\;,\\ 0\;\;\text{otherwise}\;. \end{array}\right. \end{array}$$ (29)

On the basis of above reconstruction, a semi discrete high resolution kinetic solver is formulated as

$$\begin{array}{ccc} \frac{dW_{i}}{dt} & = & -\frac{F_{i+\frac{1}{2}}\left(W_{i+1}^{LX},W_{i}^{RX}\right)-F_{i-\frac{1}{2}}\left(W_{i}^{LX},W_{i-1}^{RX}\right)}{\Delta x}\\ & & -\frac{T_{i+\frac{1}{2}}\left(W_{i+1}^{LX},W_{i}^{RX}\right)-T_{i-\frac{1}{2}}\left(W_{i}^{LX},W_{i-1}^{RX}\right)}{\Delta x}\;. \end{array}$$ (30)

In the above equation,

$$\begin{array}{c} F_{i+\frac{1}{2}}\left(W_{i+1}^{LX},W_{i}^{RX}\right)=F_{i}^{+}\left(W_{i}^{RX}\right)+F_{i+1}^{-}\left(W_{i+1}^{RX}\right)\;. \end{array}$$ (31)

To obtain second order accuracy in time, a second order total variation diminishing (TVD) Runge-Kutta (R-K) scheme is used to solve Eq (30). Let us denote the right-hand side of Eq (30) as *L*(*W*). A second order TVD R-K scheme updates *W* through the following two stages [22]

$$\begin{array}{c} W^{\left(1\right)}=W^{n}+\Delta tL\left(W^{n}\right)\;, \end{array}$$ (32)

$$\begin{array}{c} W^{n+1}=\frac{1}{2}\left(W^{n}+W^{\left(1\right)}+\Delta t\;L\left(W^{\left(1\right)}\right)\right)\;, \end{array}$$ (33)

where, Δ*t* = *t*^*n*+1^ − *t*^*n*^ represents the time step. In all our 1D computations, we have chosen the dynamic time steps according to the following CFL condition:

$$\begin{array}{c} dt\leq \max_{1\leq i\leq N}\left(\frac{dx}{2|\sigma_{i}|}\right), \end{array}$$ (34)

where *σ*_*i*_ is the maximum eigenvalue of the Jacobin matrix A(W) given by Eq (11).

## Single-phase two-dimensional Ripa model

Now, we consider the two-dimensional Ripa system in which the water temperature fluctuations are taken into account. The two dimensional Ripa model has the following form

$$\begin{array}{c} \partial_{t}h+\partial_{x}\left(hu\right)+\partial_{y}\left(hv\right)=0\;, \end{array}$$ (35)

$$\begin{array}{c} \partial_{t}\left(hu\right)+\partial_{x}\left(hu^{2}+\frac{g}{2}h^{2}\theta \right)+\partial_{y}\left(huv\right)=-gh\theta \partial_{x}B\;, \end{array}$$ (36)

$$\begin{array}{c} \partial_{t}\left(hv\right)+\partial_{x}\left(huv\right)+\partial_{y}\left(hv^{2}+\frac{g}{2}h^{2}\theta \right)=-gh\theta \partial_{y}B\;, \end{array}$$ (37)

$$\begin{array}{c} \partial_{t}\left(h\theta \right)+\partial_{x}\left(uh\theta \right)+\partial_{y}\left(vh\theta \right)=0\;. \end{array}$$ (38)

Here, *h* represents the water height, *u* and *v* are respectively the fluid velocities in the *x* and *y* directions, *B* = *B*(*x*, *y*) denotes the bottom topography and the gravitational constant is represented by *g*. The variable *θ* is referred to as the potential temperature field and *hu* and *hv* are the momentums along *x* and *y* directions, respectively. The stationary steady states for this system are

$$\begin{array}{c} u=v=0,\;w=h+B=\text{constant},\;\theta =\text{constant}. \end{array}$$ (39)

In compact form, the above system can be rewritten as

$$\begin{array}{c} \frac{\partial W}{\partial t}+\frac{\partial F(W)}{\partial x}+\frac{\partial G(W)}{\partial y}=\tau ,\;\; \end{array}$$ (40)

where W is the vector of conserved variable, *F*(*W*) and *G*(*W*) represent fluxes along *x*-axis and *y*-axis respectively, and *τ* denotes the vector of source terms. The conserved variables are given as

$$\begin{array}{c} w_{1}\overset{\text{def}}{=}h,\;w_{2}\overset{\text{def}}{=}hu,\;w_{3}\overset{\text{def}}{=}hv,\;w_{4}\overset{\text{def}}{=}h\theta \; \end{array}$$ (41)

and the conservative fluxes are expressed as

$$\begin{array}{c} f_{1}\overset{\text{def}}{=}hu,\;f_{2}\overset{\text{def}}{=}hu^{2}+\frac{g}{2}h^{2}\theta ,\;f_{3}\overset{\text{def}}{=}huv,\;f_{4}\overset{\text{def}}{=}hu\theta \;, \end{array}$$ (42)

$$\begin{array}{c} g_{1}\overset{\text{def}}{=}hv,\;g_{2}\overset{\text{def}}{=}huv,\;g_{3}\overset{\text{def}}{=}hv^{2}+\frac{g}{2}h^{2}\theta ,\;g_{4}\overset{\text{def}}{=}hv\theta \;. \end{array}$$ (43)

Moreover, the non-conservative source terms are defined as

$$\begin{array}{c} \tau_{1}\overset{\text{def}}{=}0,\;\tau_{2}\overset{\text{def}}{=}-gh\theta \frac{\partial B}{\partial x},\;\tau_{3}\overset{\text{def}}{=}-gh\theta \frac{\partial B}{\partial y},\;\tau_{4}\overset{\text{def}}{=}0. \end{array}$$ (44)

The quasi-linear form of the above system is as follows

$$\begin{array}{c} \partial_{t}W+H\left(W\right)\partial_{x}\left(W\right)+J\left(W\right)\partial_{y}\left(W\right)=S\left(W\right)\;, \end{array}$$ (45)

where *W* = (*w*_1_, *w*_2_, *w*_3_, *w*_4_)^*T*^, *S*(*W*) = [0, −*ghθ*∂_*x*_ *B*, −*ghθ*∂_*y*_ *B*, 0]^*T*^. *H*(*W*) and *J*(*W*) are the Jacobian matrices of the system under consideration. The matrices *H*(*W*) and *J*(*W*) are given as

$$\begin{array}{c} H\left(W\right)=\left(\begin{array}{cccc} 0 & 1 & 0 & 0\\ -u^{2}+\frac{g}{2}h\theta & 2u & 0 & \frac{g}{2}h\\ -uv & v & u & 0\\ -u\theta & \theta & 0 & u\; \end{array}\right),\;J\left(W\right)=\left(\begin{array}{cccc} 0 & 0 & 1 & 0\\ -uv & v & u & 0\\ -v^{2}+\frac{g}{2}h\theta & 0 & 2v & \frac{g}{2}h\\ -v\theta & 0 & \theta & v\; \end{array}\right). \end{array}$$ (46)

Eigenvalues of the Jacobian matrix H(W) are *α*_1_ = *α*_2_ = *u*, $\alpha_{3}=u+\sqrt{gh\theta }$ and $\alpha_{4}=u-\sqrt{gh\theta }$. Similarly, the eigenvalues of the Jacobian matrix J(W) are *β*_1_ = *β*_2_ = *v*, $\beta_{3}=v+\sqrt{gh\theta }$ and $\beta_{4}=v-\sqrt{gh\theta }$. As the eigenvalues of two-dimensional Ripa system are all real, so it is a hyperbolic system having independent eigenvectors. To calculate the time step of the numerical scheme, eigenvalues are needed in the time step relation for ensuring the stability of the scheme. If we take $u=v=\pm \sqrt{gh\theta }$, the Jacobian of the Ripa system will not provide a complete set of eigenvalues, i.e. the Ripa system produces a resonance phenomenon. For that reason, the solution of the Riemann problems becomes very difficult in such situations. The current KFVS for the Ripa system avoids such complexities because it calculates the cell interface fluxes from the Maxwellian distribution function and it does not require a Riemann solver.

## KFVS scheme for two-dimensional single-phase shallow flow model

Now consider the two-dimensional single-phase shallow flow model in Eqs (35)–(38). In this case, the flux is associated with the particles motion across the boundaries of cell interfaces. In the two-dimensional case, the flow of particles along each characteristic direction *x* and *y* is considered using the macroscopic flux functions *F*(*W*) and *G*(*W*) through boundaries of the mesh cells. For example, for flow in the *x*-direction, the flux function is determined by the particle motion in that direction. The remaining quantities, e.g. velocity in *y*–direction and water height may be treated as passive scalars which are moved with the *x*-direction particle’s velocity. The same Maxwellian distribution *f*_*M*_ in Eq (12) is considered with *n* ∈ {*x*, *y*} to split the flux functions in the *x* and *y*-directions. In the *x*-direction, we have

$$\begin{array}{c} F=F^{+}+F^{-}\;, \end{array}$$ (47)

where,

$$\begin{array}{c} F^{\pm }={\left\langle v^{1}\right\rangle }_{\pm x}\left(\begin{array}{c} h\\ hu\\ hv\\ h\theta \end{array}\right)+{\left\langle v^{0}\right\rangle }_{\pm x}\left(\begin{array}{c} 0\\ \frac{1}{2}gh^{2}\theta \\ 0\\ 0 \end{array}\right). \end{array}$$ (48)

Similarly, in the *y*-direction

$$\begin{array}{c} G=G^{+}+G^{-}\;, \end{array}$$ (49)

where,

$$\begin{array}{c} G^{\pm }={\left\langle v^{1}\right\rangle }_{\pm y}\left(\begin{array}{c} h\\ hu\\ hv\\ h\theta \end{array}\right)+{\left\langle v^{0}\right\rangle }_{\pm y}\left(\begin{array}{c} 0\\ 0\\ \frac{1}{2}gh^{2}\theta \\ 0 \end{array}\right). \end{array}$$ (50)

Let *N*_*x*_ and *N*_*y*_ be large integers in the *x* and *y*-directions, respectively. We assume a Cartesian grid with a rectangular domain [*x*_0_, *x*_max_] × [*y*_0_, *y*_*max*_] which is covered by cells *C*_*ij*_ ≔ [*x*_*i*−1/2_, *x*_*i*+1/2_] × [*y*_*j*−1/2_, *y*_*j*+1/2_] for 1 ≤ *i* ≤ *N*_*x*_ and 1 ≤ *j* ≤ *N*_*y*_. Here, (*x*_*N*_*x*_+1/2_, *y*_*N*_*y*_+1/2_) = (*x*_*max*_, *y*_*max*_) The representative coordinates in cell *C*_*ij*_ are denoted by (*x*_*i*_, *y*_*j*_). Therefore,

$$\begin{array}{c} \left(x_{1/2},y_{1/2}\right)=\left(x_{0},y_{0}\right),\;\;\;\;\left(x_{N_{x}+1/2},y_{N_{y}+1/2}\right)=\left(x_{max},y_{max}\right) \end{array}$$ (51)

$$\begin{array}{c} x_{i}=\frac{x_{i-1/2}+x_{i+1/2}}{2}\;,\;y_{j}=\frac{y_{j-1/2}+y_{j+1/2}}{2} \end{array}$$ (52)

and

$$\begin{array}{c} \Delta x=x_{i+1/2}-x_{i-1/2}\;,\;\Delta y=y_{j+1/2}-y_{j-1/2}\;. \end{array}$$ (53)

Thus, fluxes at the cell interfaces are described as

$$\begin{array}{c} F_{i+\frac{1}{2},j}=F_{i,j}^{+}+F_{i+1,j}^{-}\;,\;G_{i,j+\frac{1}{2}}=G_{i,j}^{+}+G_{i,j+1}^{-}.\; \end{array}$$ (54)

Integration of Eq (17) over *C*_*i*,*j*_ gives the following semi-discrete kinetic upwind scheme

$$\begin{array}{ccc} \frac{dW_{i,j}}{dt} & = & -\frac{F_{i+\frac{1}{2},j}-F_{i-\frac{1}{2},j}}{\Delta x}-\frac{T_{i+\frac{1}{2},j}-T_{i-\frac{1}{2},j}}{\Delta x}\\ & & -\frac{G_{i,j+\frac{1}{2}}-G_{i,j-\frac{1}{2}}}{\Delta y}-\frac{T_{i,j+\frac{1}{2}}-T_{i,j-\frac{1}{2}}}{\Delta y}, \end{array}$$ (55)

where, the cell averaged values *W*_*i*,*j*_ are defined as

$$\begin{array}{c} W_{i,j}:=W_{i,j}\left(t\right)=\frac{1}{\Delta x\Delta y}\int_{x_{i-\frac{1}{2}}}^{x_{i+\frac{1}{2}}}\int_{y_{i-\frac{1}{2}}}^{y_{i+\frac{1}{2}}}W\left(t,x,y\right)dydx\;. \end{array}$$ (56)

The cell interface fluxes $F_{i+\frac{1}{2},j}$ and $G_{i,j+\frac{1}{2}}$ are defined by Eq (54). Moreover, $T_{i+\frac{1}{2},j}$ − $T_{i-\frac{1}{2},j}$ and $T_{i,j+\frac{1}{2}}$ − $T_{i,j-\frac{1}{2}}$ are expressed as

$$\begin{array}{c} T_{i+\frac{1}{2}}-T_{i-\frac{1}{2}}=\left(\begin{array}{c} 0\\ -gh\theta [{\left(B\right)}_{i+\frac{1}{2}}-{\left(B\right)}_{i-\frac{1}{2}}]\\ 0\\ 0 \end{array}\right)\;, \end{array}$$ (57)

$$\begin{array}{c} T_{j+\frac{1}{2}}-T_{j-\frac{1}{2}}=\left(\begin{array}{c} 0\\ 0\\ -gh\theta [{\left(B\right)}_{j+\frac{1}{2}}-{\left(B\right)}_{j-\frac{1}{2}}]\\ 0 \end{array}\right)\;. \end{array}$$ (58)

The same reconstruction procedure can be applied in direction by direction manner which was discussed in Eqs (26)–(29) of the previous section. Therefore, the final form of the 2D-KFVS scheme is given as

$$\begin{array}{ccc} \frac{dW_{i,j}}{dt} & = & -\frac{F_{i+\frac{1}{2},j}\left(W_{i+1,j}^{LX},W_{i,j}^{RX}\right)-F_{i-\frac{1}{2},j}\left(W_{i,j}^{LX},W_{i-1,j}^{RX}\right)}{\Delta x}\\ & & -\frac{T_{i+\frac{1}{2},j}\left(W_{i+1,j}^{LX},W_{i,j}^{RX}\right)-T_{i-\frac{1}{2},j}\left(W_{i,j}^{LX},W_{i-1,j}^{RX}\right)}{\Delta x}\\ & & -\frac{G_{i,j+\frac{1}{2}}\left(W_{i,j+1}^{LY},W_{i,j}^{RY}\right)-G_{i-\frac{1}{2},j}\left(W_{i,j}^{LY},W_{i,j-1}^{RY}\right)}{\Delta y}\\ & & -\frac{T_{i,j+\frac{1}{2}}\left(W_{i,j+1}^{LY},W_{i,j}^{RY}\right)-T_{i-\frac{1}{2},j}\left(W_{i,j}^{LY},W_{i,j-1}^{RY}\right)}{\Delta y}. \end{array}$$ (59)

The second order TVD RK scheme (Eqs (32) and (33)) is used to solve the system of ordinary differential equations in Eq (59). In all our 2D computations, we have chosen the dynamic time steps according to the following CFL condition:

$$\begin{array}{c} dt\leq 0.5\min(\frac{dx}{\max_{1\leq i\leq N_{x},1\leq j\leq N_{y}}\left(\right|\sigma_{i,j}^{x}\left|\right)},\frac{dy}{\max_{1\leq i\leq N_{x},1\leq j\leq N_{y}}\left(\right|\sigma_{i,j}^{y}\left|\right)}), \end{array}$$ (60)

where $\sigma_{i,j}^{x}$ and $\sigma_{i,j}^{y}$ are the maximum eigenvalues of the Jacobian matrices *H*(*W*) and *J*(*W*) given by Eq (46). The proposed KFVS schemes for 1D and 2D flows can be analogously extended to the 3D flows. In that case, the same Maxwellian distribution can be used to split the flux functions along the characteristic directions x, y and z. For example, for flow in the x-direction, the flux function is determined by the particles motion in that direction. The remaining quantities, such as velocities in the y and z directions and water height may be treated as passive scalars moving with x-direction velocity of particles. Moreover, the current KFVS scheme can be easily extended to solve multidimensional incompressible and compressible multiphase flow models, see for example [39, 46].

There are so many techniques and procedures which can be used to further enhance the accuracy and reduce the computational cost of the above KFVS schemes. For example one can increase the order of accuracy by using WENO-limiters and can apply adaptive meshing techniques to reduce the computational cost [47]. One can also adopt Singh’s numerical scheme for further improvements [48]. Moreover, one can also try to establish a variational formulation for the discussed systems [49].

## Numerical case studies

In this section, several case studies are carried out in one and two-space dimensions. The initial data of these Riemann problems contain a single discontinuity. There are conditions, called as “shock conditions”, which decide the occurrence of shocks. These conditions can be used to find the state on the other side of the shock if a state on the one side of the shock is already known [4, 50]. The results obtained from KFVS scheme are compared with the results of central NT scheme. The main reason for choosing NT scheme is its simplicity, robustness and efficiency. Moreover, the scheme does not requires any exact or approximate Riemann solver. If we want to apply a Godunov upwind scheme then we have to derive the exact Riemann solver and a complete set of eigenvalues which are very difficult or almost impossible. The KFVS scheme is applied for the first time to the Ripa system incorporating variable bottom topography and horizontal temperature gradients. In all the test problems outflow boundary conditions are used.

### One-dimensional case studies

Here, we present the one-dimensional test problems. In the first problem, a variable bottom topography and smooth initial data are considered to calculate *L*^1^-errors and *L*^∞^-errors of both schemes. The next two test problems are related to the flat bottom and the last three problems involve non-flat bottom topography. The initial data for each case study are given along with it. The reference solutions are obtained on 2000 grid points. Both KFVS and central schemes give identical solutions at refined grids. Thus, either central scheme or KFVS scheme can be used to obtain reference solutions. In all 1D results the black solid line represents the reference solution, the black dashed line denotes the numerical solution of KFVS scheme, and the gray dashed line is used to represent the numerical solution of central NT scheme. In error plots KFVS scheme is represented by black dotted line and grey dotted line denotes the central NT scheme. The value of gravitational constant is taken as *g* = 1 in all test problems.

#### Problem 1: Convergence study of KFVS scheme

This problem is taken from [24] to study the convergence of proposed numerical scheme for a smooth solution. The computational domain is chosen as [−1, 1] and the bottom topography function in the form of a smooth bump is defined as

$$\begin{array}{c} B\left(x\right)=\{\begin{array}{cc} 2.0(\cos(10\pi x)+1), & \text{if}\;-0.1\leq x\leq 0.1,\\ 0, & \text{otherwise.} \end{array} \end{array}$$ (61)

The initial data are given as

$$\begin{array}{c} \left(h,u,\theta \right)=\begin{array}{c} (3+\exp(0.1x),\exp(0.1x),2\exp(0.1x)). \end{array} \end{array}$$ (62)

**Fig 1:**
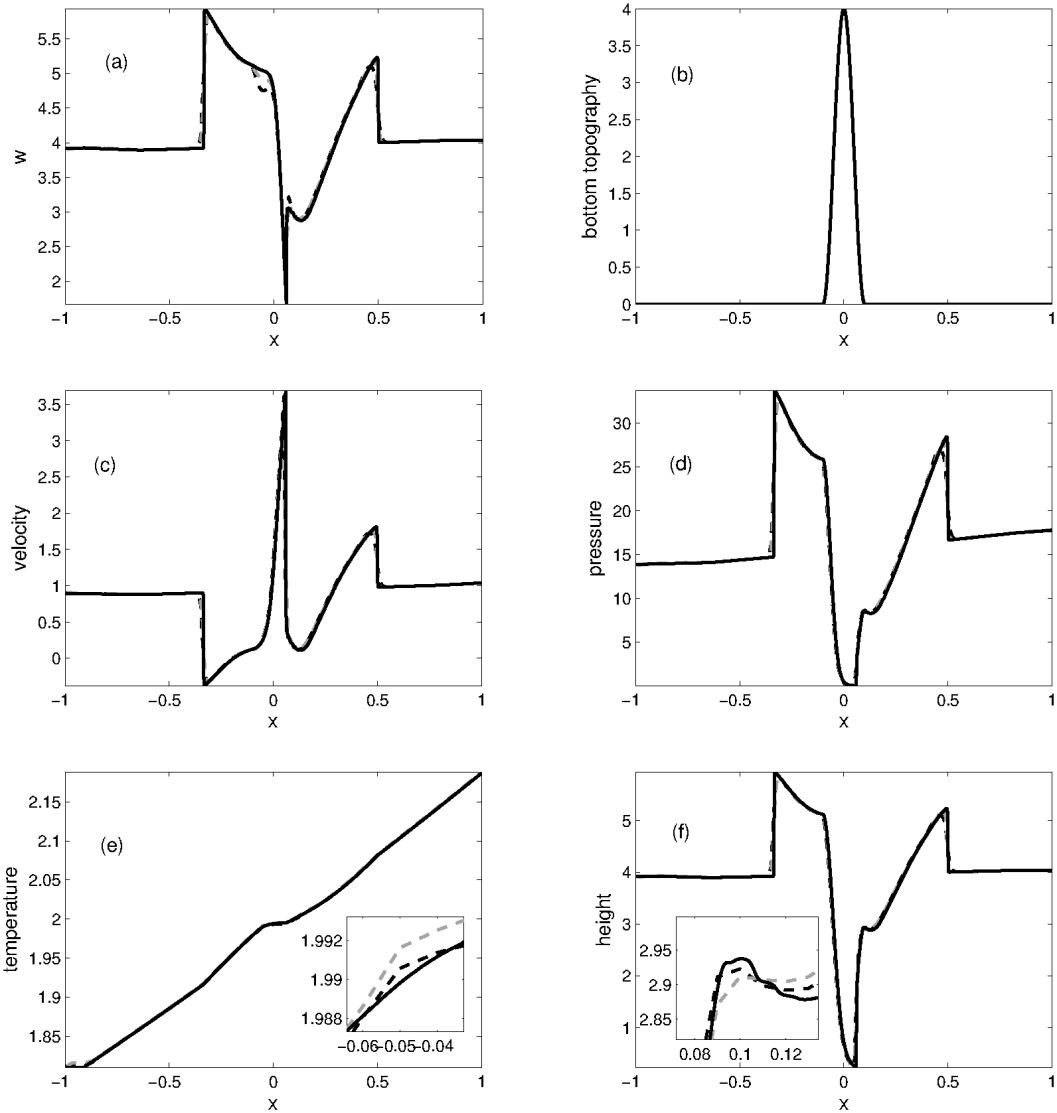
Problem 1: A comparison of KFVS and NT central scheme results at time *t* = 0.1.

**Fig 2:**
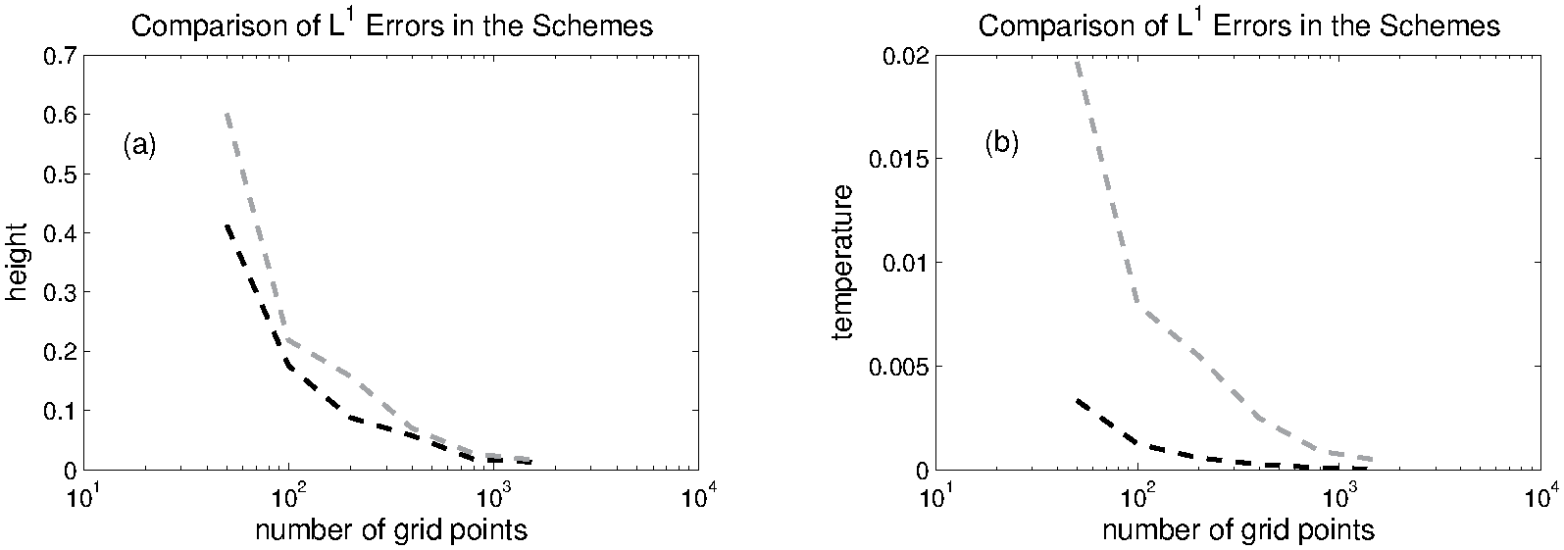
Problem 1: A comparison of *L*^1^ errors in the height and temperature of both schemes at different grid points.

**Fig 3:**
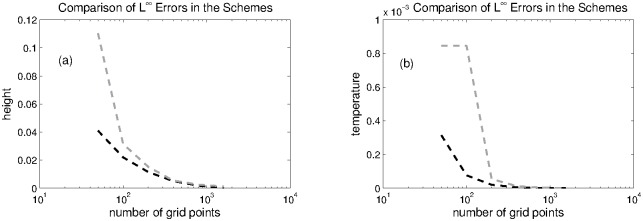
Problem 1: A comparison of *L*^∞^ errors in height and temperature of both schemes at different grid points.

The computational domain is divided into 200 grid points on which numerical solutions are calculated. The numerical solutions at *t* = 0.1 are plotted in Fig 1. The *L*^1^ and *L*^∞^ errors are computed for water height and temperature with respect to reference solution, which are displayed in the Tables 1 and 2, respectively. The *L*^1^-errors and *L*^∞^-errors in both schemes are functions of grid points which are also ploted in Figs 2 and 3. It can be observed that KFVS method produces less errors in the solutions and has faster convergence rate when the number of grid points are increased.

**Table 1:** Comparison of *L*^1^ errors in both schemes.

N	Height (*h*)		Temperature (*θ*)	
	KFVS	Central	KFVS	Central
50	0.356751	0.600986	0.003329	0.019655
100	0.174911	0.218685	0.001234	0.007957
200	0.080786	0.157829	0.000574	0.005498
400	0.034853	0.070073	0.000252	0.002494
800	0.013600	0.026827	0.000088	0.000895
1600	0.002903	0.016336	0.000016	0.000434

**Table 2:** Comparison of *L*^∞^ errors in both schemes.

N	Height (*h*)		Temperature (*θ*)	
	KFVS	Central	KFVS	Central
50	0.041013	0.110370	0.000314	0.000846
100	0.021939	0.031463	0.000076	0.000221
200	0.011476	0.015185	0.000020	0.000054
400	0.004947	0.005351	0.000005	0.000012
800	0.001743	0.002517	0.000001	0.000002
1600	0.000229	0.001190	0.000000	0.000001

In the literature, the accuracy and efficiency of the KFVS scheme has been analyzed and verified for several nonlinear hyperbolic systems, see for example [21–23, 31, 38, 39, 41, 46].

#### Problem 2: Dam break over the flat bottom

This dam break problem over the flat bottom was considered in [7]. Here, we have solved it for the Ripa system with flat bottom topography (*B* ≡ 0). The computational domain is [−1, 1] and the initial data are given as

$$\begin{array}{c} \left(w,u,\theta \right)=\{\begin{array}{cc} (5,0,3), & \text{if}\;x<0,\\ (1,0,5), & \text{if}\;x>0. \end{array} \end{array}$$ (63)

**Fig 4:**
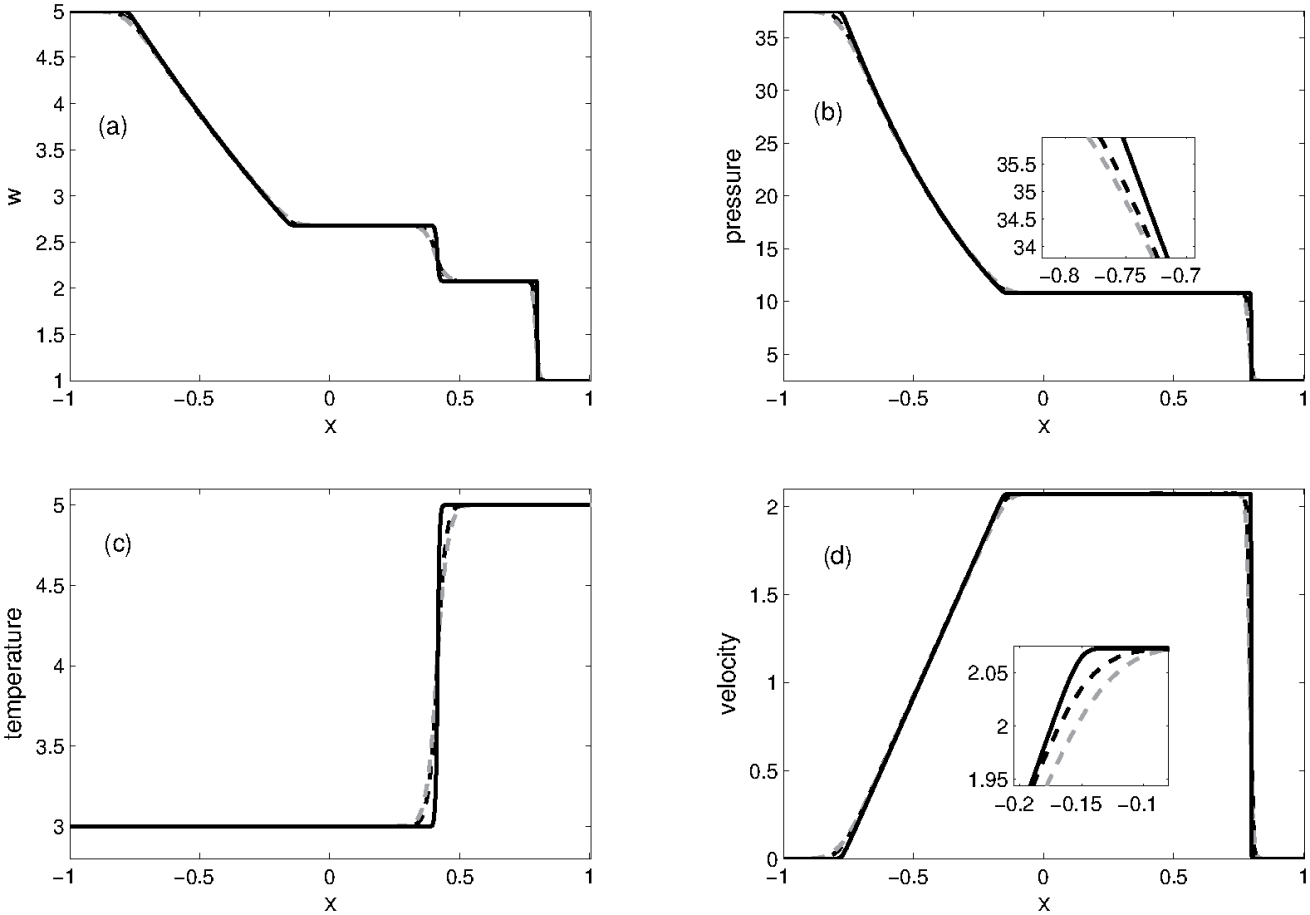
Problem 2: Dam break over the flat bottom. A comparison of KFVS scheme with NT central scheme at time *t* = 0.2.

The computational domain is divided into 200 grid points. In this problem, we have computed *w* = *h* + *B*, *u*, *θ* and *p* using the KFVS scheme and compared its results with those of central NT scheme. All the results are plotted in Fig 4 at time *t* = 0.2. It can be seen that results of both schemes are in good agreement with each other. Both KFVS and central schemes give comparable results. However, KFVS scheme gives better resolution of sharp fronts in the solutions.

#### Problem 3: 1D dam break over a flat waterbed

Our last one-dimensional flat bottom experiment is a dam break over a flat waterbed. The initial conditions for this problem are defined as [6]

$$\begin{array}{c} \left(h,u,\theta \right)=\{\begin{array}{cc} (2,0,1), & \text{if}\;\;|x|\leq 0.5,\\ (1,0,1.5), & \text{otherwise.} \end{array} \end{array}$$ (64)

**Fig 5:**
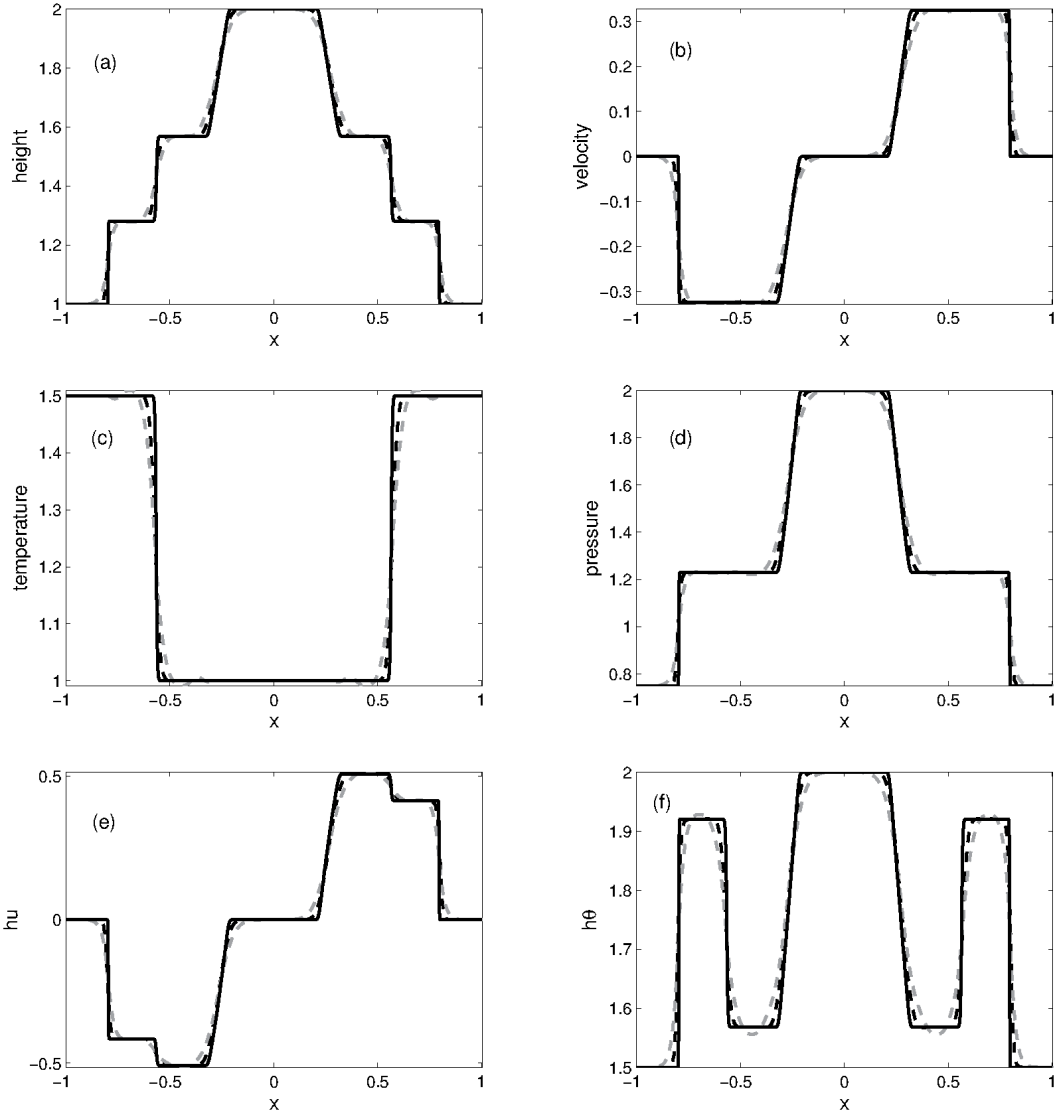
Problem 3: Dam break over the flat waterbed. A comparison of KFVS scheme with NT central scheme at time *t* = 0.2.

The numerical solution is calculated at *t* = 0.2 in the computational domain [−1, 1] using the KFVS scheme and its results are compared with those obtained from the central NT scheme. The computational domain is divided into 200 grid points. We noticed that when waterbed is flat, the source term appearing in the Ripa system vanishes and the system can be easily solved by using any finite volume scheme. Fig 5 shows the plots of *h*, *u*, *θ*, *p*, *hu* and *hθ*. A good agreement can be observed between the results of KFVS and central NT schemes.

#### Problem 4: Dam break over the non-flat bottom

This problem is also taken from [7] which we have solved for the Ripa system with non-flat bottom using the following initial data

$$\begin{array}{c} \left(w,u,\theta \right)=\{\begin{array}{cc} (5,0,1), & \text{if}\;x<0,\\ (1,0,5), & \text{if}\;x>0. \end{array} \end{array}$$ (65)

The variable bottom topography function is defined as

$$\begin{array}{c} B\left(x\right)=\{\begin{array}{cc} 2.0(\cos(10\pi (x+0.3))+1), & \text{if}\;-0.4\leq x\leq -0.2,\\ 0.5(\cos(10\pi (x-0.3))+1), & \text{if}\;0.2\leq x\leq 0.4,\\ 0 & \text{otherwise.} \end{array} \end{array}$$ (66)

**Fig 6:**
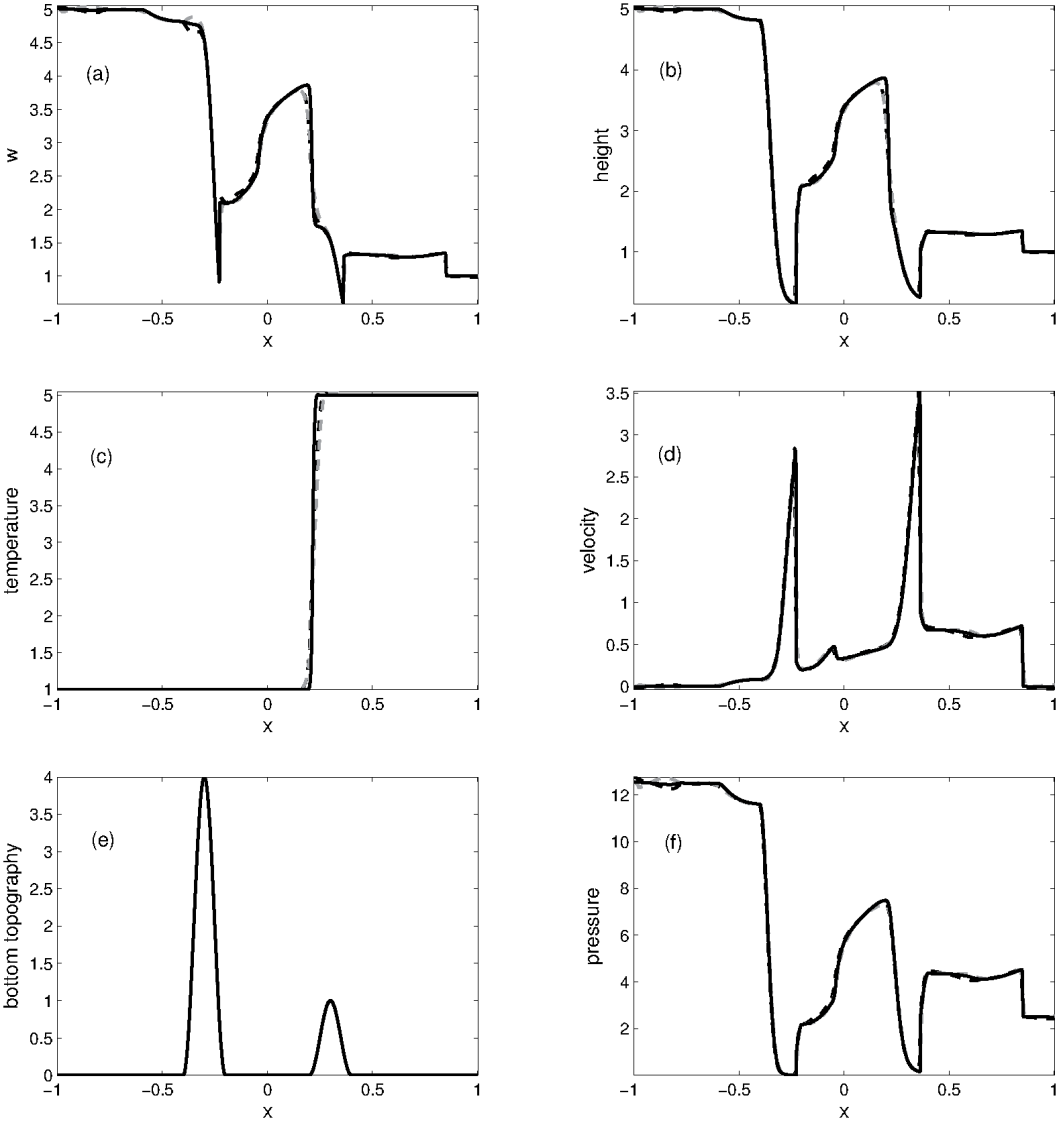
Problem 4: Dam break over the nonflat bottom. A comparison of KFVS scheme with NT central scheme at time *t* = 0.3.

This problem is solved to verify the performance of KFVS and central schemes for non-homogeneous shallow water equations. The computational domain [−1, 1] is discretized into 200 grid points. The results of numerical schemes at *t* = 0.3 are plotted in Fig 6. Initially the area near *x* = 0.3 is almost dry, as *h* = *w* − *B*(*x*), thus

$$\begin{array}{c} h(x,0)=1-0.5(\cos(10\pi (x-0.3))+1). \end{array}$$ (67)

Both the schemes are found well-balanced and preserve the positivity of *w*, *h* and *θ*.

#### Problem 5: Small perturbation of a steady state solution

This one-dimensional experiment describes a small perturbation of a steady-state solution [7]. The initial conditions are given as

$$\begin{array}{c} \left(w,u,\theta \right)=\{\begin{array}{cc} (6,0,4), & \text{if}\;x<0,\\ (4,0,9), & \text{if}\;x>0. \end{array} \end{array}$$ (68)

The non-flat bottom topography is defined as

$$\begin{array}{c} B\left(x\right)=\{\begin{array}{cc} 0.85(\cos(10\pi (x+0.9))+1), & \text{if}\;-1.0\leq x\leq -0.8,\\ 1.25(\cos(10\pi (x-0.4))+1), & \text{if}\;0.3\leq x\leq 0.5,\\ 0, & \text{otherwise.} \end{array} \end{array}$$ (69)

**Fig 7:**
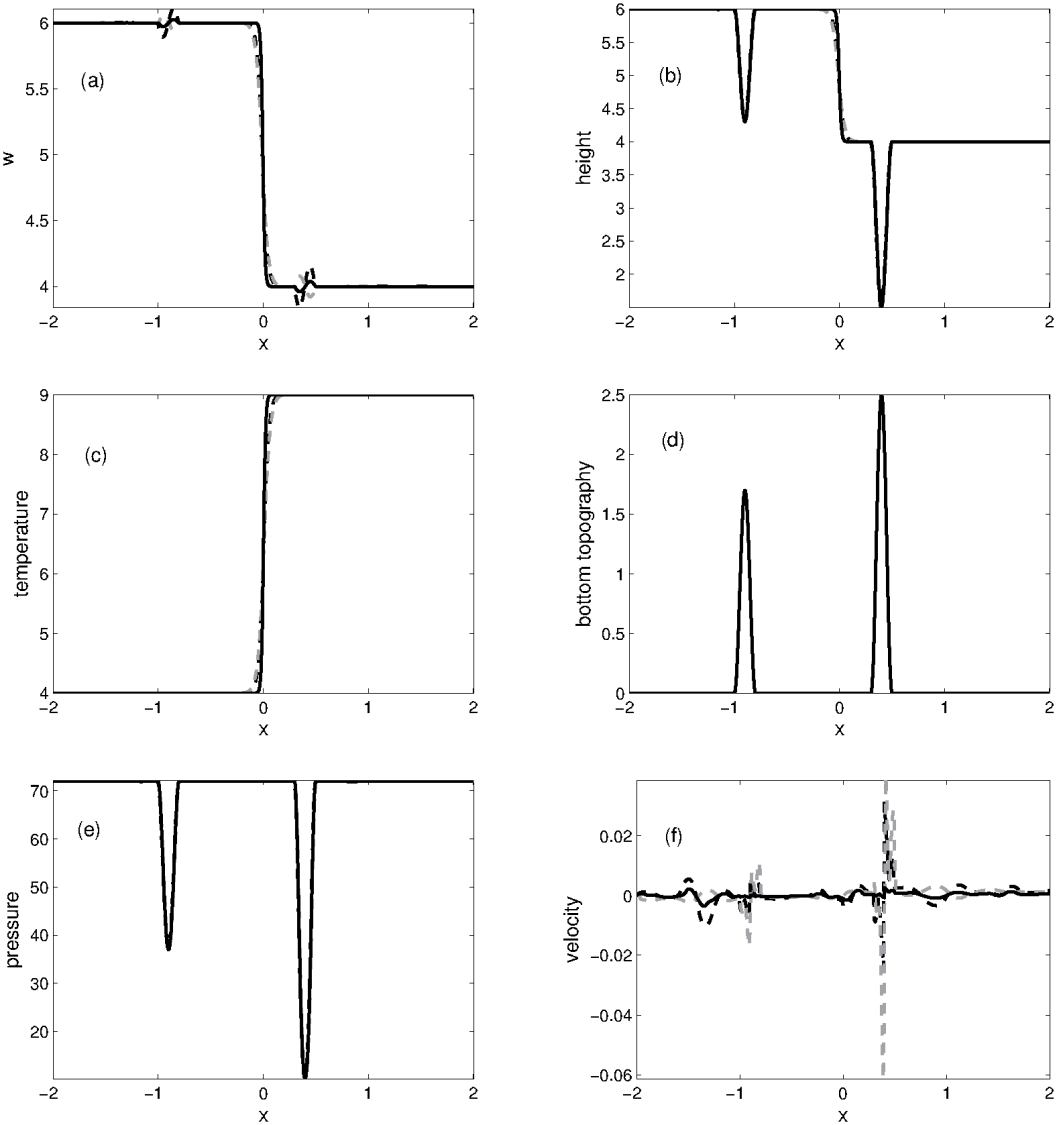
Problem 5: Small Perturbation of a steady-state solution. A comparison of KFVS scheme with NT central scheme at time *t* = 0.4.

In this problem, the numerical solutions are obtained at *t* = 0.4 in the computational domain [−2, 2]. The computational domain is divided into 200 grid points. The numerical results are presented in Fig 7. It can be observed that with an increase in time the perturbation splits into two pulses moving in opposite directions. The first right moving pulse passes through the first hump of the bottom and keeps moving over the temperature jump and then passes through the second hump of the bottom. The results obtained by KFVS scheme are better than the central NT scheme.

#### Problem 6: 1D dam break problem over a rectangular bump

Our last one-dimensional problem of variable bottom topography is a discontinuous waterbed problem which is taken from [6] for the case of Ripa system. The waterbed for the rectangular bump is defined as

$$\begin{array}{c} B\left(x\right)=\{\begin{array}{cc} 8,\; & \text{if}\;\;|x-300|<75,\\ 0, & \text{otherwise} \end{array} \end{array}$$ (70)

and the initial data are given by

$$\begin{array}{c} \left(h,u,\theta \right)=\{\begin{array}{cc} ((20-B(x),0,1), & \text{if}\;x\leq 300,\\ ((15-B(x),0,5), & \text{otherwise.} \end{array} \end{array}$$ (71)

**Fig 8:**
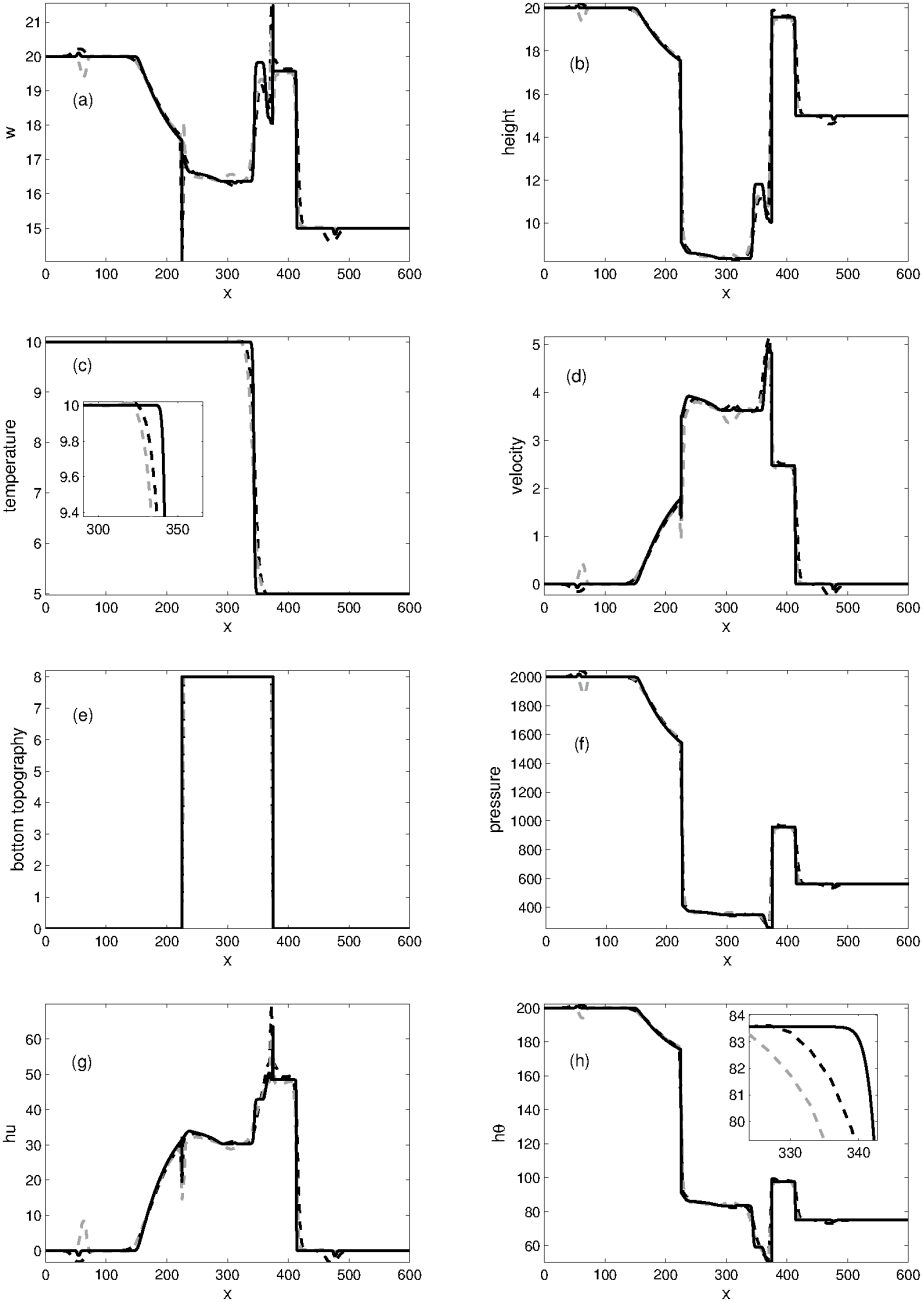
Problem 6: 1D dam break problem over the rectangular bump. A comparison of KFVS scheme with NT central scheme at time *t* = 12.

Numerical solutions are obtained in the computational domain [0, 600] at time *t* = 12 using the well-balanced KFVS and central NT schemes. The computational domain is divided into 200 grid points. The plots for *w* = *h* + *B*, *h*, *θ*, *p*, *u*, *B*, *hu* and *hθ* are given in Fig 8. Both the schemes have the ability to capture sharp variation in the solution profile. However, the KFVS scheme captures the peaks more efficiently.

### Two-dimensional case studies

In this section, four test problems are considered for the two-dimensional single-phase shallow flows on flat or non-flat bottom topographies, i.e. *B*(*x*, *y*) = 0 or *B*(*x*, *y*) ≠ 0. Here, we have discretized the computational domain [−1, 1] × [−1, 1] into 100 × 100 mesh cells for the numerical solutions. Moreover, the 2D results obtained by the KFVS scheme are compared with those obtained from the central NT scheme and their comparison is expressed in the 1D form. In the 1D plots, the results of KFVS scheme are represented by black solid lines and those of central NT scheme are denoted by dashed grey lines.

#### Problem 7: Rectangular dam break problem

This two-dimensional classical rectangular dam break problem over the flat bottom was considered in [6]. The two constant states of initial conditions are given as

$$\begin{array}{c} \left(h,u,v,\theta \right)=\{\begin{array}{cc} (2,0,0,1), & \text{if}\;\;|x|\leq 0.5,\\ (1,0,0,1.5), & \text{otherwise}. \end{array} \end{array}$$ (72)

**Fig 9:**
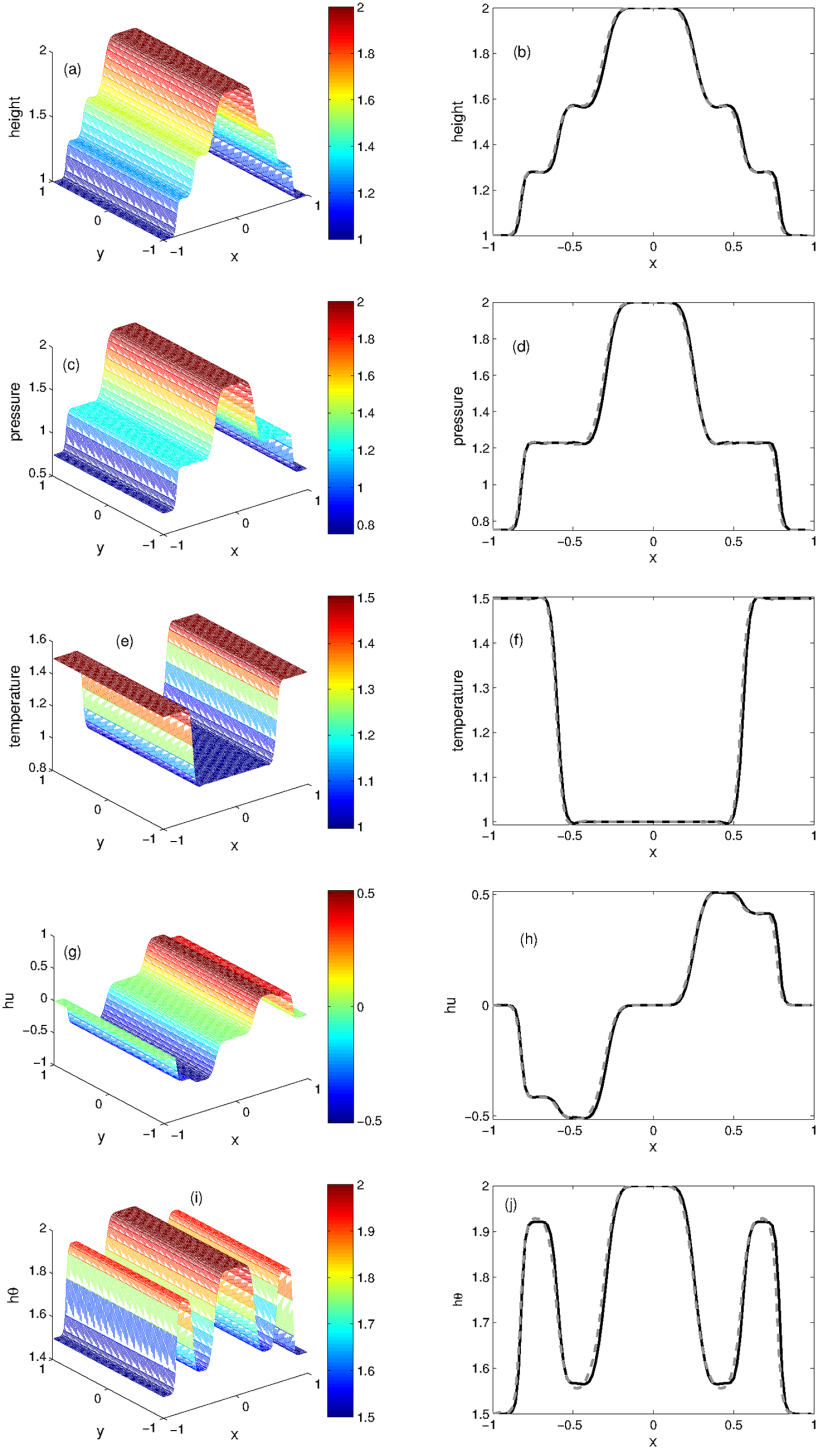
Problem 7: Rectangular dam break problem. 3D views of solution components(left) and their 1D comparison(right) computed by KFVS and central NT schemes at time *t* = 0.2 using uniform mesh Δx = Δy = 2/100.

The numerical solutions are computed in the computational domain [−1, 1] at the final simulation time *t* = 0.2 using the well balanced KFVS and central NT schemes. The 3D plots (left) of KFVS scheme and their comparison with the central scheme in the form of 1D plots (right) are presented in Fig 9. The solutions obtained show two shock and two contact waves moving away from the vertical axis and two rarefaction waves propagating towards vertical axis. A good agreement can be seen between the results of both schemes. However, KFVS is more efficient and accurate.

#### Problem 8: Radial dam break over the flat bottom

This circular dam break over a flat bottom was considered in [7] for the shallow flow model. We have solved this problem for the Ripa system using the following initial data

$$\begin{array}{c} \left(w,u,v,\theta \right)=\{\begin{array}{cc} (2,0,0,1), & \text{if}\;x^{2}+y^{2}<0.25,\\ (1,0,0,1.5), & \text{otherwise}. \end{array} \end{array}$$ (73)

**Fig 10:**
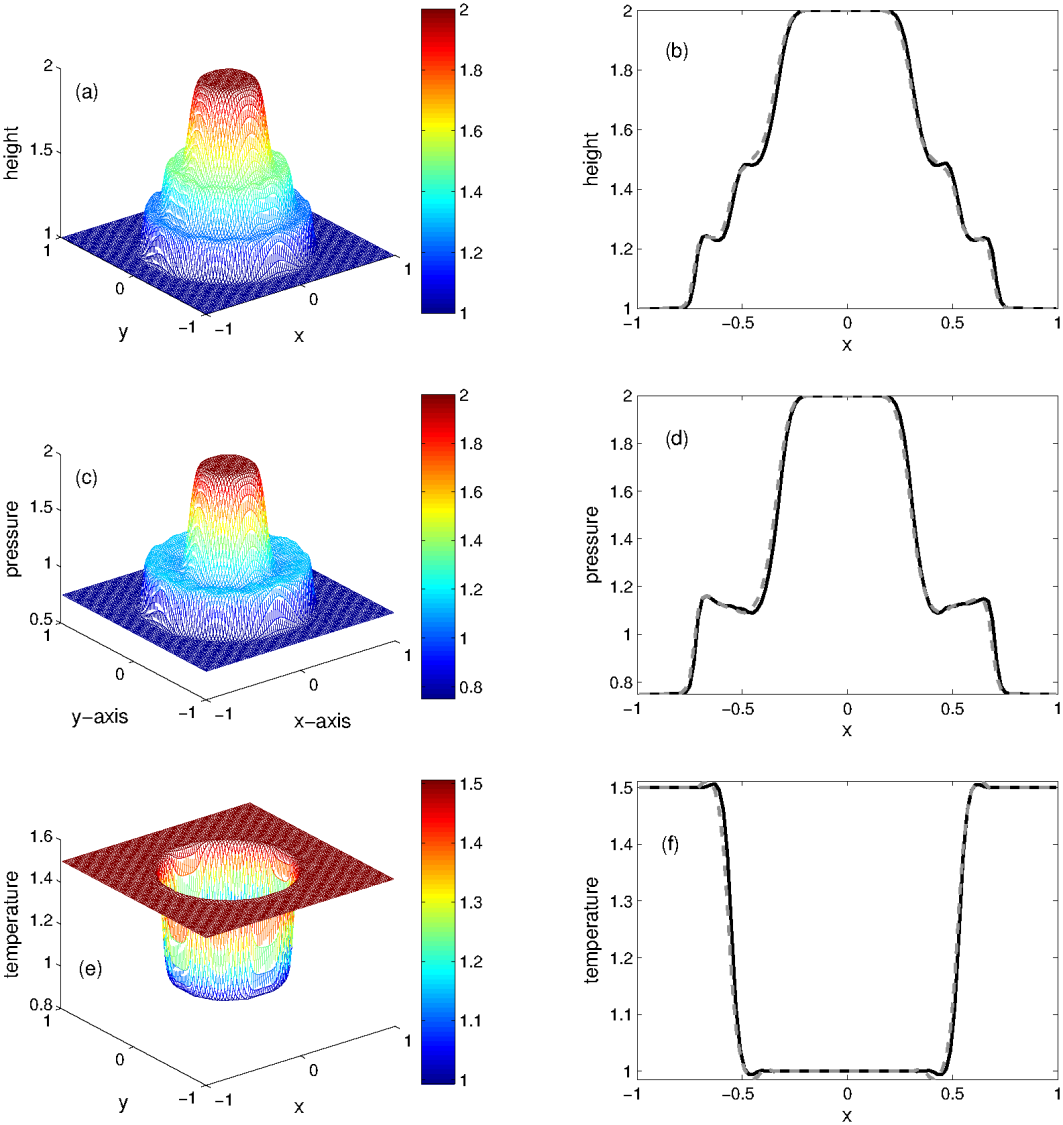
Problem 8: Radial dam break problem over the flat bottom. 3D views of solution components(left) and their 1D comparison(right) computed by KFVS with central NT schemes at time *t* = 0.15 using uniform mesh Δx = Δy = 2/100.

**Fig 11:**
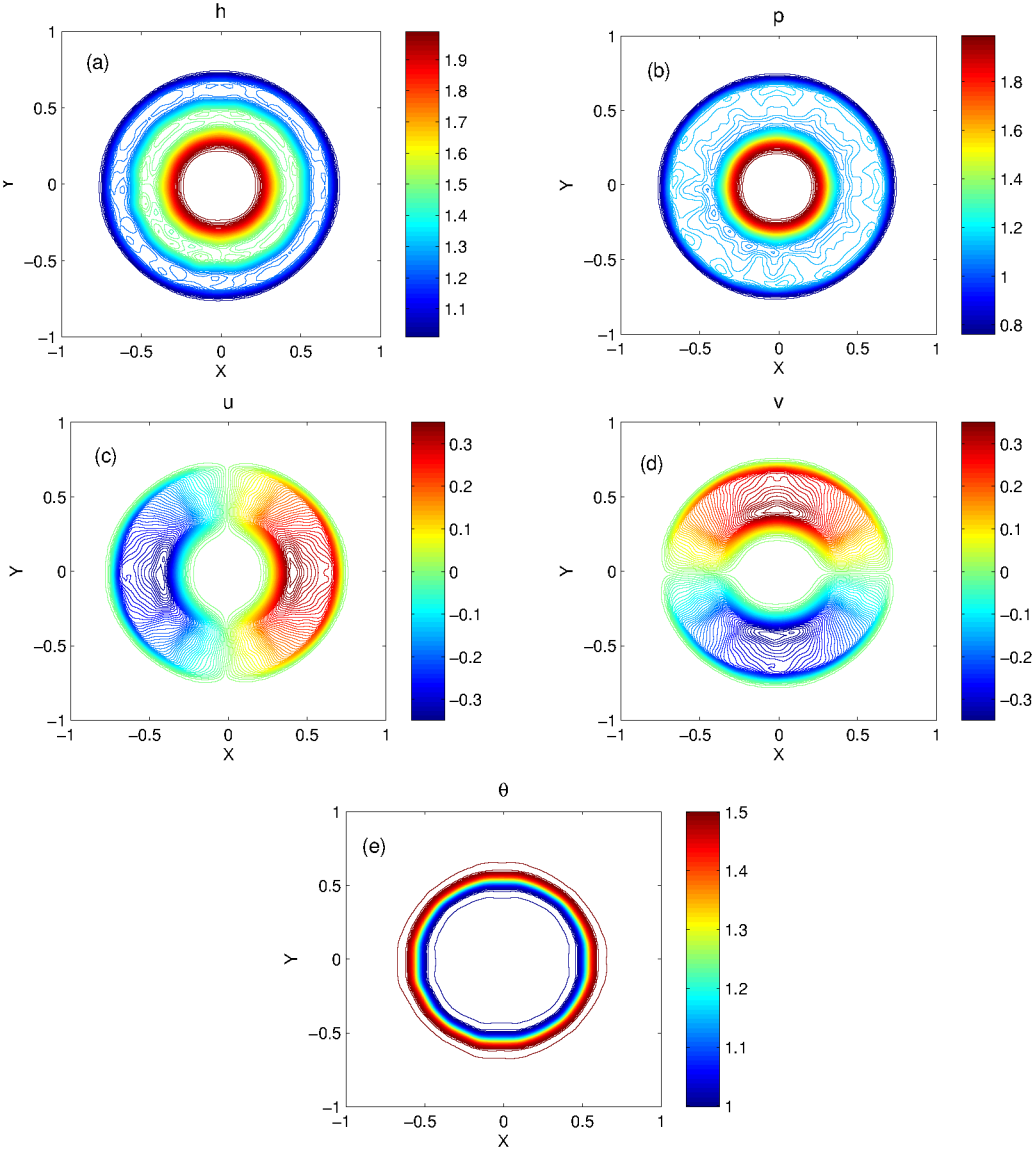
Problem 8: Radial dam break problem over the flat bottom. Contour plots of solution components computed by KFVS scheme at time *t* = 0.15 using uniform mesh Δx = Δy = 2/100.

In this experiment, we compare the results of KFVS and central schemes for flat bottom topography. The 3D plots of KFVS method and the 1D plots for comparison of the two schemes are shown in Fig 10 at *t* = 0.15 using the uniform mesh Δ*x* = Δ*y* = 0.02. When the dam breaks, a shock wave is generated which moves radially outwards, a rarefaction wave travels radially inward and a contact wave moves between the shock and rarefaction waves. One can easily see that a good agreement is found between both schemes. It is observed that some small oscillations are generated in the contact area, which can be seen in the contour plots of KFVS scheme presented in Fig 11.

#### Problem 9: Perturbation of steady states on an irregular waterbed

This 2D steady state problem was considered for Ripa system in [6, 7]. It contains a bottom topography function having two Gaussian shaped humps. The initial conditions, featuring the steady states of a lake at rest are given below

$$\begin{array}{c} \left(w,u,v,\theta \right)=\{\begin{array}{cc} (3,0,0,\frac{4}{3}), & \text{if}\;x^{2}+y^{2}<0.25\\ (2,0,0,3), & \text{otherwise} \end{array} \end{array}$$ (74)

and the variable waterbed is defined as follows

$$\begin{array}{c} B\left(x,y\right)=\{\begin{array}{cc} 0.5exp[-100\left({\left(x+0.5\right)}^{2}+{\left(y+0.5\right)}^{2}\right)], & \text{if}\;x\leq 0,\\ 0.6exp[-100\left({\left(x-0.5\right)}^{2}+{\left(y-0.5\right)}^{2}\right)], & \text{if}\;x>0. \end{array} \end{array}$$ (75)

**Fig 12:**
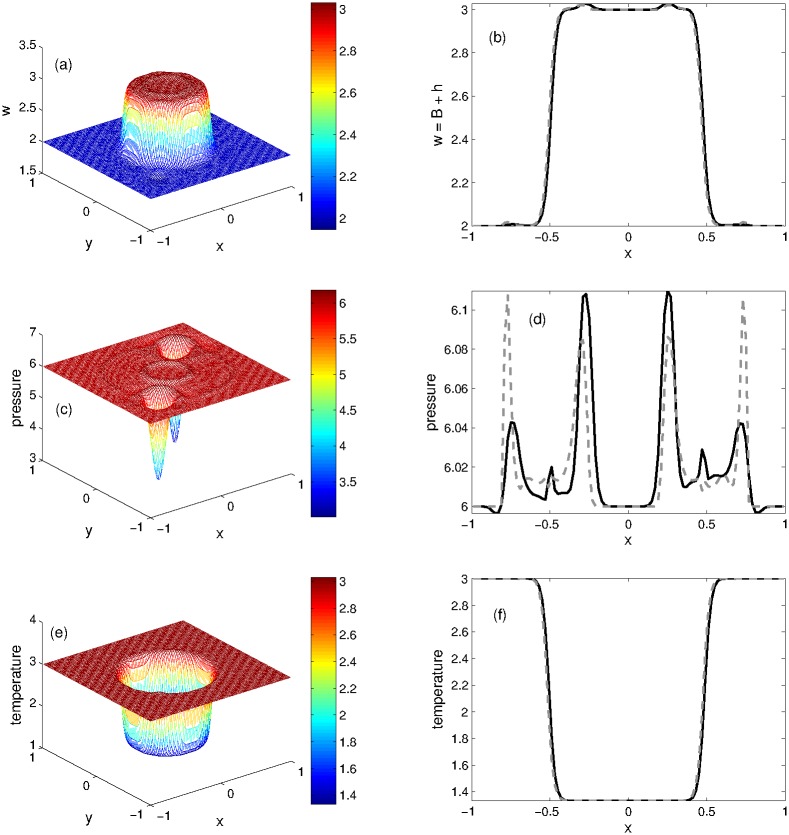
Problem 9: Perturbation of steady states on an irregular waterbed. 3D views of solution components(left) and their 1D comparison(right) computed by KFVS and central NT schemes at time *t* = 0.12 using uniform mesh Δx = Δy = 2/100.

**Fig 13:**
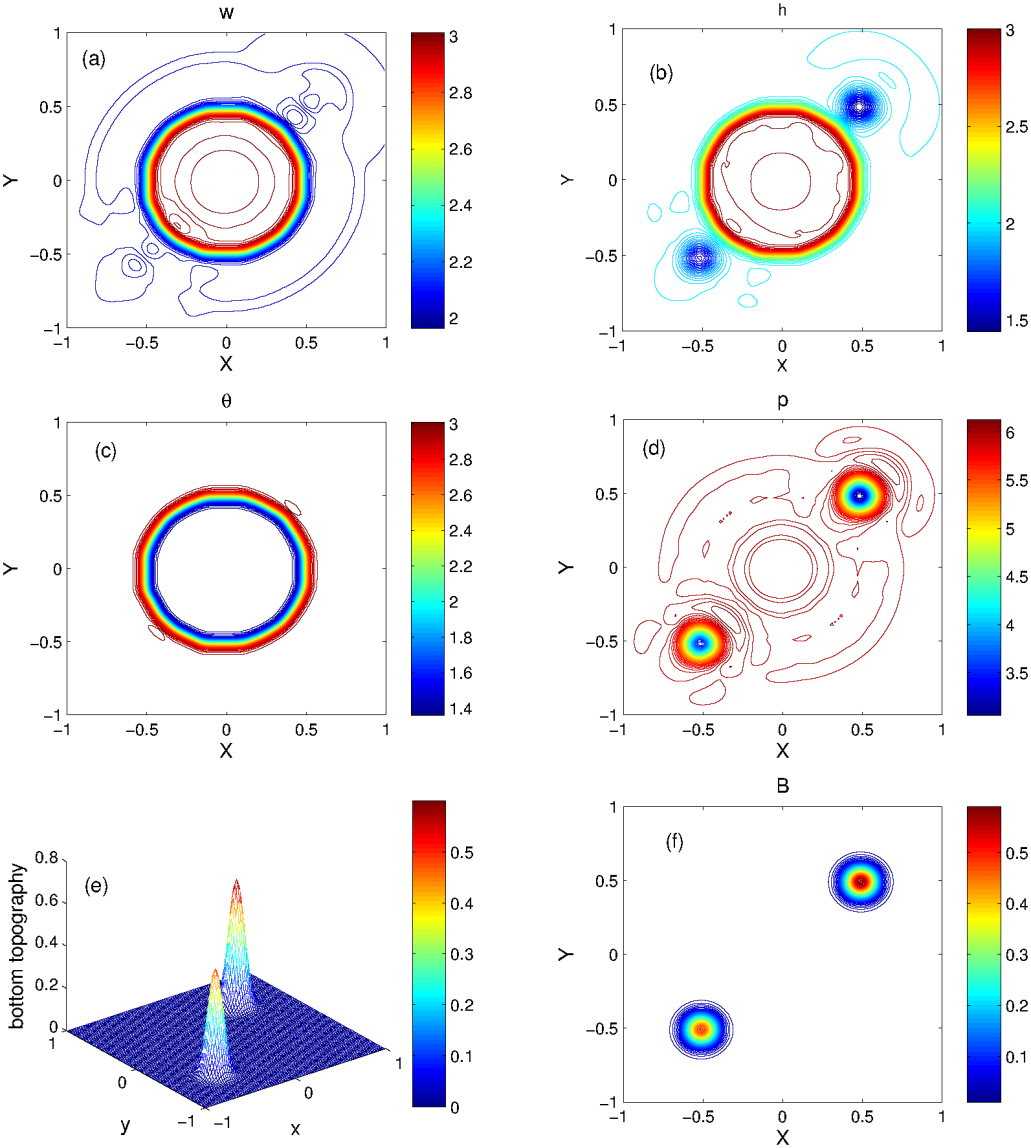
Problem 9: Perturbation of steady states on an irregular waterbed. Contour plots of solution components computed by KFVS scheme at time *t* = 0.12 using uniform mesh Δx = Δy = 2/100.

The steady state numerical solution is perturbed by introducing the bottom topography and water height. The numerical results of KFVS and central NT schemes are computed at *t* = 0.12. The 3D plots of KFVS scheme and the 1D plots for the comparison of schemes are given in Fig 12. One can easily see from the plots that the proposed KFVS scheme preserves the steady states of *w*, *θ* and radial pressure oscillations. Moreover, the contours of KFVS scheme for this problem are presented in Fig 13.

#### Problem 10: Small perturbation of steady states solution

In this case study, we use the same bottom topography as defined in the Problem 10, but initial data are perturbed near the center of the computational domain [−1, 1] inside the two small annulus [0.01, 0.09] and [0.09, 0.25] for two humps. The initial data for this problem are given as

$$\begin{array}{c} \left(w,u,v,\theta \right)=\{\begin{array}{cc} (3.1,0,0,\frac{4}{3}), & \text{if}\;0.01<x^{2}+y^{2}<0.09,\\ (3,0,0,\frac{4}{3}), & \text{if}\;0.09<x^{2}+y^{2}<0.25,\\ (2,0,0,3), & \text{otherwise}. \end{array} \end{array}$$ (76)

**Fig 14:**
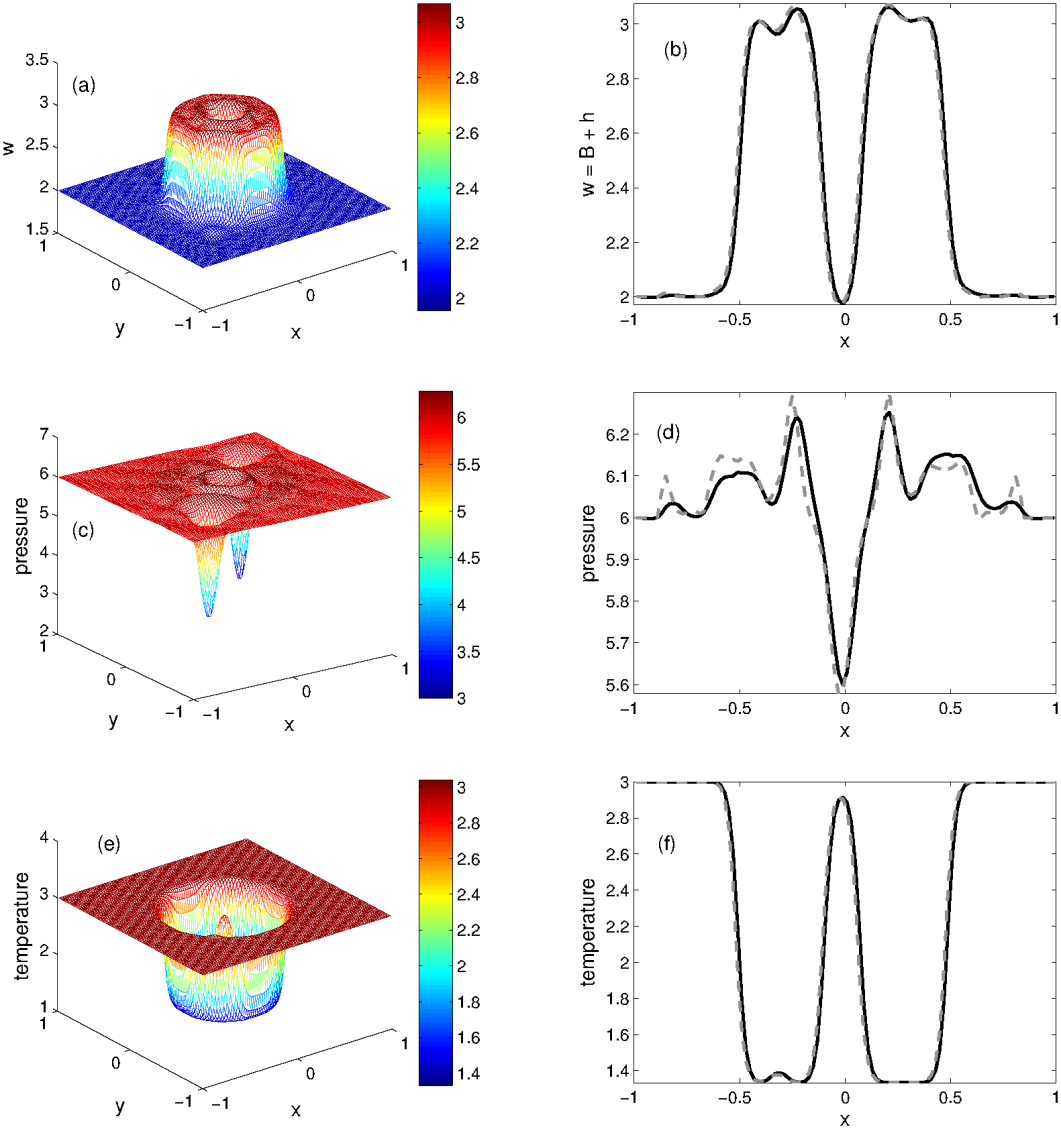
Problem 10: Small perturbation of a steady states solution. 3D views of solution components(left) and their 1D comparison(right) computed by KFVS and central NT schemes at time *t* = 0.15 using uniform mesh Δx = Δy = 2/100.

**Fig 15:**
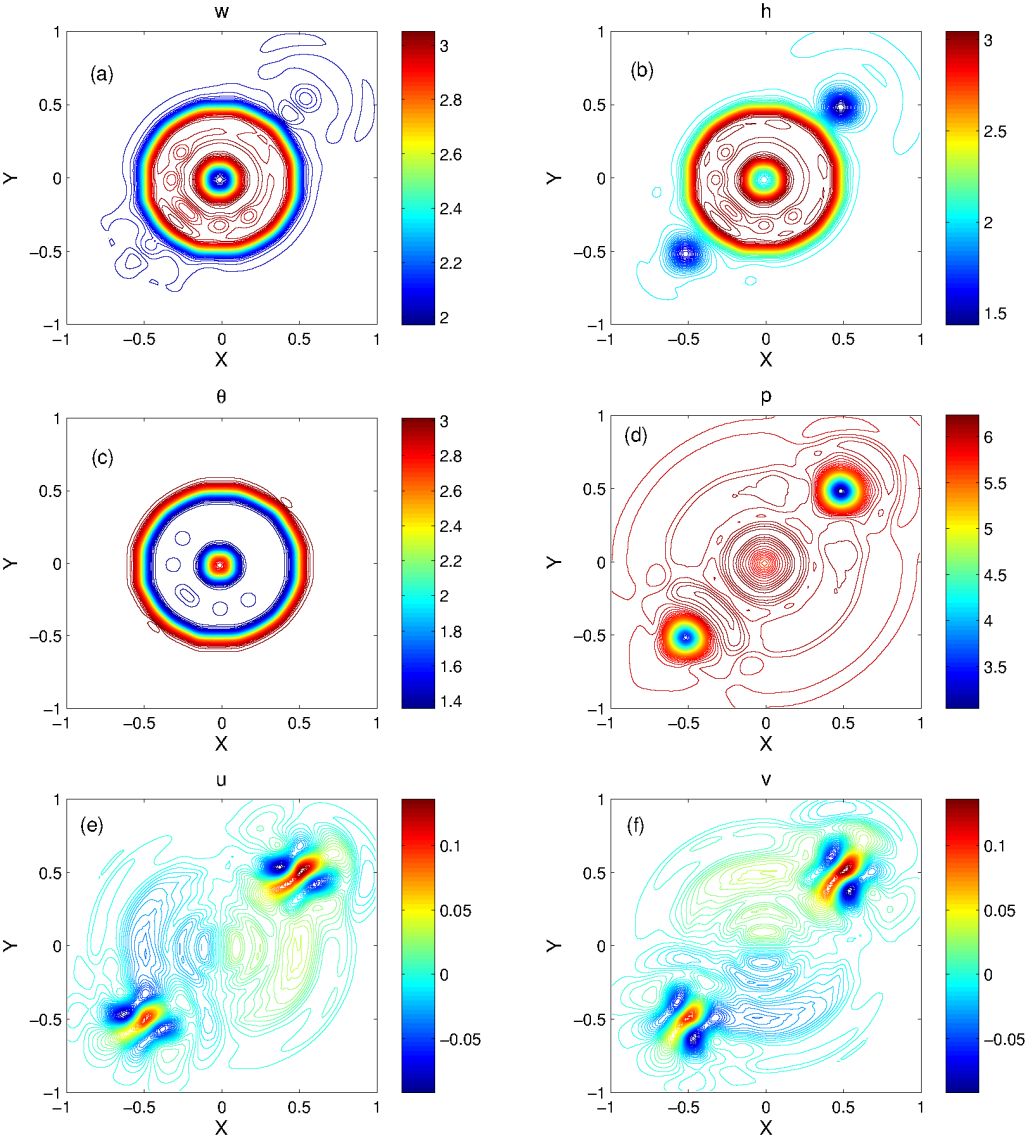
Problem 10: Small perturbation of a steady states solution. Contour plots of solution components computed by KFVS scheme at time *t* = 0.15 using uniform mesh Δx = Δy = 2/100.

The numerical solutions are obtained at *t* = 0.15 and perturbation propagation can be seen in the plots given in Figs 14 and 15. The 3D plots of KFVS scheme and the 1D plots for comparisons of both schemes are given in Fig 14. The solution obtained from central scheme develops radial shaped small oscillations in the pressure solution, which interact with the perturbation. Due to the interaction of circular shape pressure oscillations and perturbation, parasitic waves appear in the contour of pressure that can be seen easily in Fig 15. Moreover, KFVS scheme shows its capability to capture perturbation more accurately.

## Conclusion

In this paper, we have developed a high resolution KFVS scheme to approximate the one and two-dimensional Ripa systems. The KFVS method was applied for the first time to solve the Ripa systems for both flat and non-flat bottom topographies. The proposed scheme has the ability to capture sharp discontinuities, efficiently handles dry bed regions, and is extendable for multi-phase flow problems. The scheme preserves the non-negativity of the flow height and temperature. To satisfy the well-balanced nature of the scheme for the Ripa system, we have discretized the source term analogously to the discretization of flux divergence terms. The scheme was validated by taking into account some classical test problems already available in the recent literature. The results of KFVS scheme were compared with those obtained from the staggered central NT scheme and the reference solutions. The numerical results of both schemes were found in good agreement with each other and those available in the literature. However, it was observed that KFVS gives better resolution of sharp discontinuities as compared to the central scheme. The scheme has preserved the well-balanced property and non-negativity in the problems of steady sate perturbed variable waterbed. This confirms the efficiency of the KFVS method to accurately solve the one and two dimensional Ripa systems. The considered numerical simulations include the dam-break problems, small perturbation problems of variable bottom topography, and varying temperature gradients.
